# Drivers of northern peatland CO_2_ fluxes revisited: interacting water level-temperature dependency

**DOI:** 10.1038/s41467-026-77456-6

**Published:** 2026-09-04

**Authors:** Nicolas Behrens, Christian Brümmer, Kuno Kasak, June Skeeter, Ian B. Strachan, Ype van der Velde, Chris D. Evans, Ross Morrison, Carole Helfter, Guillaume Bertrand, Sébastien Gogo, Adrien Jacotot, Carsten Schaller, Karen Yeung, Mana Gharun

**Affiliations:** 1 Institute of Landscape Ecology, University of Münster, Münster, Germany; 2 Thünen Institute of Climate-Smart Agriculture, Braunschweig, Germany; 3 Department of Geography, University of Tartu, Tartu, Estonia; 4 Department of Environmental Science, Policy, and Management, University of California, Berkeley, USA; 5 Natural Resources Canada, Vancouver, Canada; 6 Department of Geography and Planning, Queen’s University, Kingston, Canada; 7 Earth and Climate, Vrije Universiteit, Amsterdam, Netherlands; 8 UK Centre for Ecology and Hydrology, Bangor, UK; 9 UK Centre for Ecology & Hydrology, Wallingford, UK; 10 UK Centre for Ecology & Hydrology, Edinburgh, UK; 11 Chrono-Environnement, UMR 6249, Université Marie et Louis Pasteur, CNRS, Montbéliard, France; 12 ECOBIO, UMR 6553, Université de Rennes, CNRS, Rennes, France; 13 Chrono-Environnement, UMR 6249, Université Marie et Louis Pasteur, CNRS, Besançon, France; 14 SARL SEMBIOSE, Saint-Denis-de-Gastines, France; 15 Lower Saxony Water Management, Coastal and Nature Protection Agency (NLWKN), Hanover, Germany

**Keywords:** Wetlands ecology, Environmental impact, Climate-change mitigation, Carbon cycle

## Abstract

Peatlands are the largest terrestrial stores of organic carbon, but drainage has turned them into substantial sources of CO_2_. While water level is widely recognized as the primary control of CO_2_ emissions from peatlands, effective future management requires understanding its interaction with rising temperatures under a warming climate. Using 276 site-years of annual CO_2_ flux observations across temperate and boreal peatlands, we apply explainable machine-learning to disentangle the combined effects of water table depths and temperature on ecosystem CO_2_ exchange at annual scales. Across peatlands spanning diverse land-cover—including natural fens and bogs, croplands, grasslands, and extraction sites—CO_2_ emissions exhibit a non-linear response to water table depth. Emissions decline when water tables are raised above 60-75 cm depth. Optimal mitigation requires water tables of 20 cm or higher. We find that deep water tables interact with temperatures to increase emissions at temperate sites. On a subset of 113 site-years of daily CO_2_ flux data, we show that at warm temperatures, higher water tables suppress, whereas deeper water tables enhance temperature-driven CO_2_ emissions from peatlands. We demonstrate that hydrology regulates the temperature sensitivity of peatland carbon release, revealing a key control on carbon–climate feedbacks under future warming.

## Abstract (web summary)

Across 276 site-years from 114 boreal and temperate peatlands, annual CO2 fluxes have a strong, nonlinear relationship with water levels. On the daily and annual scale hydrology and temperature interact, increasing emissions at warm, dry conditions.

## Introduction

Peatlands are the largest terrestrial stores of organic carbon, containing an estimated 600 Gt C^1^ while covering only 3% of the Earth’s land surface, with roughly 87% of this area located in temperate and boreal regions^2^. In their pristine, waterlogged state, oxygen limitation slows decomposition, allowing peat-forming vegetation to accumulate and store carbon over millennia. However, around 10% of global peatlands have been degraded by drainage for peat extraction, forestry, or agriculture^2^, and in densely populated regions such as central Europe, nearly all peatlands have been disturbed^3^. Drainage exposes peat to oxygen, accelerating microbial decomposition and releasing CO_2_—estimated to account for 2–5% of total anthropogenic greenhouse gas (GHG) emissions^2,4,5^. Recognizing this, initiatives such as the EU Nature Restoration Law have set targets to implement restoration measures in 30% of drained agricultural peatlands by 2030^6^. Rewetting peatlands to raise and stabilize the water table near the surface is widely regarded as the most effective measure to reduce CO_2_ emissions and curb their climate impact^7^. Yet the future trajectory of these emissions will depend not only on the extent and success of rewetting efforts but also on their interaction with rising temperatures and increasingly extreme climate conditions^8,9^. A detailed understanding of how CO_2_ emissions respond to hydrological and thermal controls is essential for predicting and managing peatland carbon dynamics in a changing climate.

To analyze and predict CO_2_ emissions from peatlands, empirical and process-based models establish functional relationships between CO_2_ fluxes and environmental drivers such as effective water table depth (the depth of the aerated peat column, WTDe) and air temperature (TA). Most existing approaches emphasize the dominant role of hydrology, proposing either linear^10,11^ or nonlinear^12,13^ relationships between WTDe and CO_2_ emissions. A proposed linear relationship suggests that partial rewetting could substantially reduce CO_2_ emissions^10^ whereas field observations indicate that CO_2_ fluxes correlate with WTDe only to a certain threshold, beyond which further lowering has little additional effect^14–17^. At the same time, peatland carbon dynamics are increasingly influenced by climate change through rising temperatures and more frequent droughts^18–20^. Site-level studies have shown that increased TA amplifies CO_2_ losses, particularly when water tables are lowered^21^, suggesting an interactive effect of hydrological and thermal controls. Yet, this interaction remains poorly quantified at larger scales, limiting the capacity to predict the feedback of carbon emissions from peatlands with different hydrological conditions under future warming.

In this study, we quantify how WTDe, TA, and their interaction affect peatland CO_2_ fluxes while accounting for additional drivers such as land-cover type, solar radiation, and leaf area. We compiled a unique dataset of 276 site-years of annual CO_2_ budgets (spanning 1999–2023) from published studies and flux networks across temperate and boreal peatlands covering major land-cover types. Explicitly, the land-cover types are bogs, fens, extraction sites, croplands and grasslands. All further mention of croplands or grasslands refers to peatlands converted for grass or crop cultivation (see “Methods”). Peatlands drained for forestry were excluded as plantation cycles span over several decades, and carbon cycles are not considered to be in a steady state^10^. The data includes budgets measured with the eddy covariance method (177 site-years) and (automatic) chambers (99 site-years). For each site, we assembled corresponding WTDe, TA, short-wave radiation (SWIN), and remotely sensed leaf area index (LAI). We applied an explainable machine-learning (XML) framework to identify key predictors and their nonlinear effects. To capture key processes that may be obscured at the annual scale, we analyzed a subset of 19 peatland sites (113 site-years) for which high-frequency CO_2_ fluxes from eddy-covariance (EC) were available in open networks or through contact with the PIs. The daily-scale analysis allows us to detect thresholds in the WTDe–CO_2_ relationship and evaluate how warm and dry conditions jointly intensify emissions during critical periods within the annual cycle^22,23^. By integrating annual and daily perspectives, we show how hydrological and thermal conditions together regulate peatland CO_2_ fluxes and identify thresholds beyond which warming and drying together increase carbon losses—information essential for improving emission estimates and guiding rewetting and restoration strategies under climate change.

## Results and discussion

### Water table depth response functions, and their limits

**Fig. 1:**
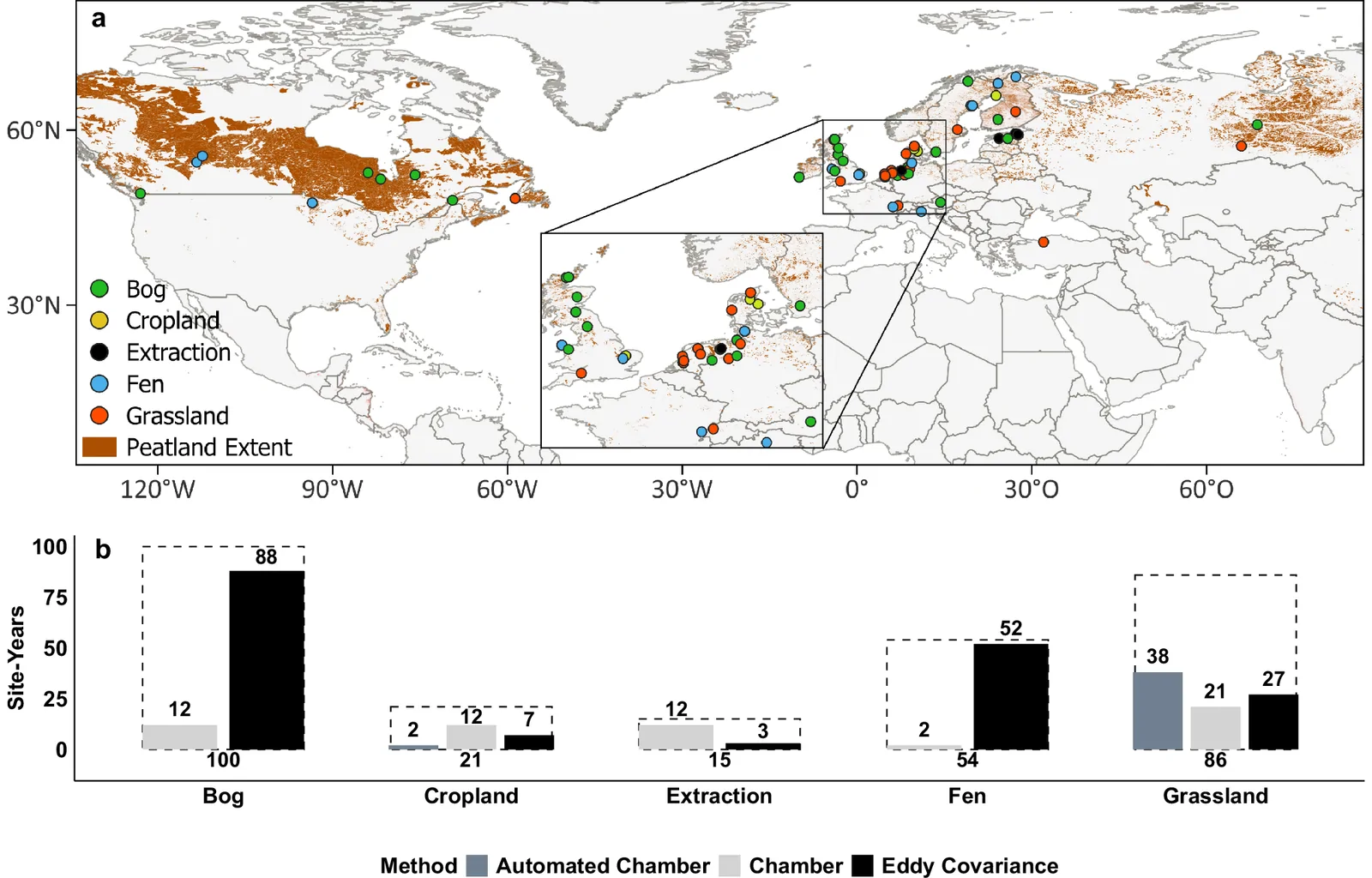
Map of peatland sites and distribution of measurement methods used to collect CO_2_ Fluxes. Location (**a**) and measurement technique (**b**) of CO_2_ fluxes at 114 compiled peatland sites used in this study (276 site-years in total). The brown shading in the map (**a**) shows the extent of peatlands in the northern hemisphere^90^. Colored dots display the location of peatland sites included in the dataset of annual net biome exchange. Numbers above bars in **b** show the number of site-years for each method, and the numbers below bars display the total number of site-years for this land-cover type.

**Fig. 2:**
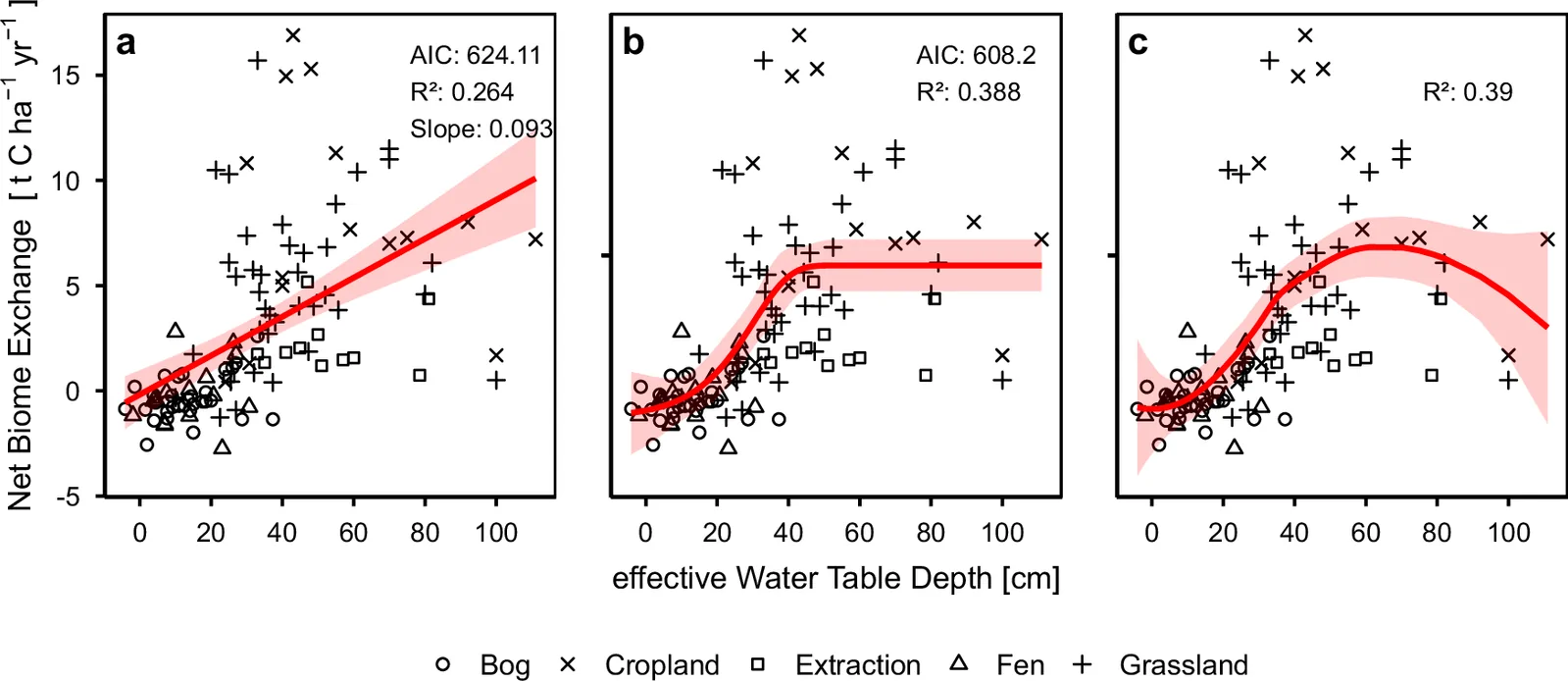
Nonlinear models fit the relationship between effective water table depth and net biome exchange better than a linear model. Comparison of linear (**a**), Gompertz (**b**), and LOESS (**c**) models of 114 mean annual Net Biome Exchange (NBE) budgets (eddy covariance and chamber data) based on the effective water table depth (WTD_e_). Shaded areas show 95% confidence intervals. Performance metrics are the proportion of explained variance (*R*^2^) and the Akaike Information Criterion (AIC).

We used data from 114 peatland sites (Fig. 1) to examine the shape and strength of the relationship between annual net biome exchange (NBE) and WTDe. NBE represents the CO_2_ flux plus biomass removal through harvest^24,25^, excluding lateral carbon fluxes such as dissolved organic carbon (DOC), since DOC measurements are not consistently available at all sites. A positive NBE refers to net CO_2_ emissions; negative values denote net CO_2_ uptake. Across peatlands representing most major land-cover types, we detected strong links between WTDe and NBE (see below). Nonlinear models generally outperformed linear ones. However, *R*^2^ of the models did not exceed 0.39 for any model based solely on WTDe (Fig. 2).

A significant linear slope (*P* < 0.001) between annual NBE and WTDe across all peatland sites (one datapoint per site) yielded an *R*^2^ of 0.27 (Fig. 2a). This is considerably lower than the 0.68 and 0.90 reported in a previous study based on EC data^10^. Our dataset included more sites (114 compared to 65), especially deeply drained ones such as grasslands, croplands and extraction sites (62 compared to 14) by utilizing new data and budgets based on chamber measurements. The weaker model performance when including more deeply drained sites challenged the suitability of a linear model to accurately represent CO_2_ fluxes across all hydrological conditions. Adding mean annual TA and satellite-based LAI as additional predictors in a multiple linear regression model (MLR) only slightly increased performance (*R*^2^ of 0.3 compared to 0.26, and AIC of 580 compared to 624). To make full use of the data of 276 site-years, we also fitted a linear mixed-effects model taking the site into account as a random intercept. The relationship between NBE and WTDe remained highly significant (*P* < 0.001). However, WTDe marginally explained only a small portion of the variation in NBE (marginal *R*^2^ = 0.14), with most of the variance attributed to differences among sites (conditional *R*^*2*^ = 0.93).

Previous incubation studies^26^ and plot-scale field experiments^15,16^ suggest that CO_2_ loss increases with declining water table depths only to a depth of 50–75 cm. To take this into account, we tested the performance of non-linear Gompertz^12,13^ and LOESS^27^ (Locally Estimated Scatterplot Smoothing) models. Both outperformed the linear and MLR models, with an *R*^2^ of 0.39 (AIC of 608 compared to 624, Fig. 2a, b). The shape and performance of the LOESS fit were similar to the Gompertz function but showed a trend of increasing emissions to a deeper WTDe of 60 cm compared to 45 cm, and a small negative trend thereafter. This pattern—where emissions peak at drainage depths of 40–80 cm before declining—confirmed observations from previous studies^12,13,17^.

The discontinuity of the linear trend may have arisen from several factors. In cropland, grassland, and extraction sites, high CO_2_ emissions can occur even at shallow WTDe (less than 50 cm from the surface). Actively managed sites are influenced by a range of practices that alter CO_2_ emissions independent of water table depth or climate. These include crop and plant selection, harvesting regimes and fertilizer application, which may affect both heterotrophic and autotrophic respiration^28–33^. In contrast, some deeply drained sites exhibited comparatively low emissions at 70–90 cm WTDe (<2.5 t C ha^−1^ yr^−1^; Fig. 2). Deep peat layers are more recalcitrant and typically show lower rates of CO_2_ production^33–37^; therefore, their aeration may contribute less to emissions. At long-term drained sites, the topmost peat layers may also be decomposed or removed, both leading to more recalcitrant peat in the upper layers^37^. Moreover, CO_2_ production was shown to peak at an optimal water-filled pore space and decline when too dry, thus water limitation may have directly reduced emissions in strongly drained sites^38–40^.

In our dataset, chamber-derived NBE in grasslands and croplands was larger than EC-derived data (Fig. S1). To address the influence of measurement methods or management, we repeated the analysis on three subsets: (1) NBE of non-harvested sites only, (2) measurements done with EC only, and (3) net ecosystem exchange (NEE) instead of NBE, which is the CO_2_ budget excluding the harvested biomass (fluxes only). Using only non-harvested sites, the performance of the linear model was similar to that of the non-linear model, and none performed significantly better than the other based on the Akaike information criterion (linear and nonlinear model having *R*^2^ of 0.39 and 0.43 and AIC of 197.7 and 196.8 respectively; see Fig. S2). However, across unmanaged sites, only four sites had a mean annual WTDe >50 cm, underrepresenting deep drainage conditions. Consistent with this limitation, linear relationships between NBE and WTDe within individual land-cover categories were not significant except for the subcategory of bogs (Fig. S3). This highlights a broader issue: drainage depth correlates with management, limiting comparable deeply drained sites and underscoring the need for more condition-specific observations. For the other subsets (2 and 3), nonlinear models consistently outperformed linear ones (*R*^2^ in non-linear models of 0.46 vs. 0.33 in linear models for EC-only, 0.39 vs. 0.19 for NEE, Fig. S2d–i). We found heteroscedasticity in the residuals of the linear and nonlinear models (*P* < 0.01), underlining the limitations in their explanatory power. With log-transformed NBE, the Gompertz function performed better (*R*^2^ of 0.57 vs. 0.39) and resolved residual heteroscedasticity while the linear model did not improve, further supporting a nonlinear WTDe–NBE relationship.

### Interacting water table and temperature effect quantified by explainable machine learning

**Fig. 3:**
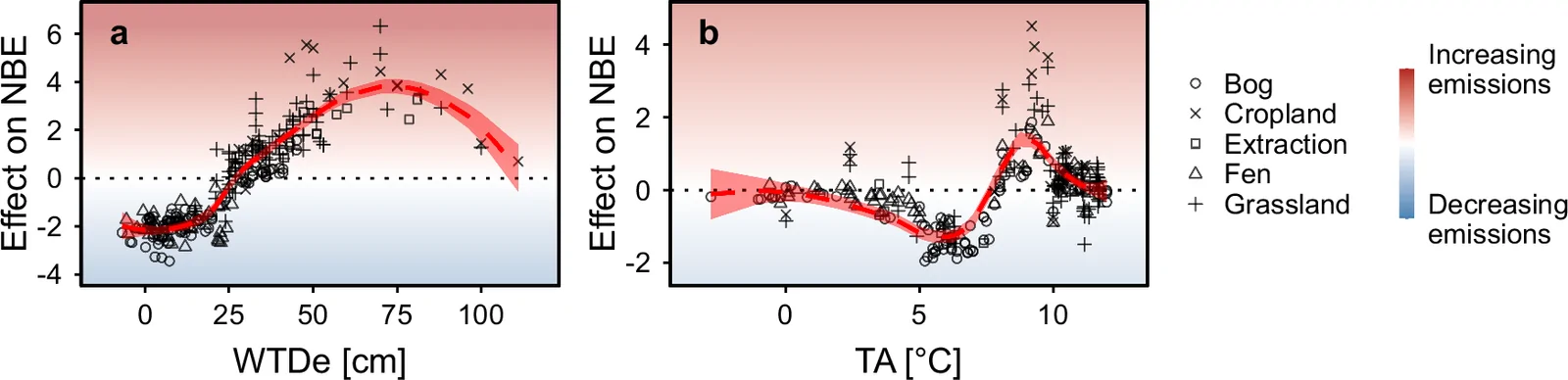
Effects of effective water table depth (WTDe) and air temperature (TA) on peatland net biome exchange (NBE) derived through explainable machine learning. Effects of WTDe (**a**) and TA (**b**) on annual NBE budgets assessed with SHAP values. SHAP values are a measure of driver strengths in machine learning models. Positive/negative SHAP values indicate an increasing/decreasing effect of the driver on NBE, while values near zero indicate a weak effect. The red dashed lines are LOESS functions, representing a smoothed response curve.

To overcome the limitations imposed by the parametric functions, we used explainable machine learning (see “Methods”), which could account for the non-linear feedback between NBE and WTDe (Fig. 3) while considering other potential predictors. We trained a machine-learning model on the full 276 site-years to predict NBE based on mean annual TA, WTDe, SWIN, LAI and the site ID and extracted the dynamics of the different drivers from it (see “Methods”). The results corroborate a nonlinear WTDe-feedback and reveal an interaction effect of WTDe with TA.

The model outperformed all regression models with an *R*^2^ of 0.71 on test data. We derived the strength and dynamics of the drivers using SHAP values, which attribute the effect of each driver on NBE for every datapoint (see “Methods”). WTDe was the main predictor with 42 ± 2% of the normalized feature importance, followed by TA (17 ± 1.7%), SWIN (15 ± 1.4%), LAI (14 ± 1.2%) and the site ID (13 ± 1.4%) with similar contributions (Fig. S4a, b). The marginal WTDe effect revealed a stronger nonlinearity, with emissions increasing until ca. 70 cm depth, compared with peaks at 45 cm to 60 cm in the regression models (Figs. 2 and  3). The transition from an emission-decreasing to an increasing effect occurred at 25–30 cm depth. The prevalence of these thresholds suggests that water tables deeper than 20–30 cm consistently lead to elevated CO_2_ emissions, while water tables need to be higher than 60–70 cm for any emission-mitigating effect to set in.

As the climate keeps warming, the role of air temperature in shaping peatland carbon fluxes is increasingly important. In our XML model, TA emerged as the second most influential predictor of NBE. Its impact was low at very cold sites (0 °C to 4 °C, Fig. 3b). We found a non-linear transition of the TA effect between sites with temperatures in the range of ca. 4 and 8 °C and above that. At the colder sites, the effect of TA on modeled NBE was small or mostly negative. At temperatures above 8 °C, the effect shifted towards a scattered but predominantly positive, NBE increasing effect, shown by largely positive SHAP values of TA. This shift was consistently found both on the full dataset (Fig. 3b) and on a dataset ensuring equal land-cover representation in warmer and colder sites (see “Methods” and Fig. S5d). Using a shallow machine learning approach, we identified 7.9 °C as the best split threshold, and the effect of TA above and below this threshold differed significantly (*P* < 0.001, see “Methods”). Based on this finding, we tested whether NBE within the landcover types differed between the colder and warmer temperature regimes, as suggested, e.g., in the IPCC Tier 1 emission factors for boreal and temperate natural peatlands^41^. The differences were non-significant for all land-cover types. The transition in SHAP-based TA effects thus rather reflects a non-linear dependency of NBE on temperature, specifically after accounting for SWIN, LAI and WTDe as well as site-specific differences, rather than a distinction of NBE budgets based on TA thresholds alone.

Because the machine-learning analysis identifies statistical relationships rather than causal mechanisms, and because the data represent annual-scale fluxes, the processes underlying the transition in TA effects cannot be directly resolved. However, a possible explanation for the shift is that warmer conditions enhance temperature-sensitive respiration more strongly than carbon uptake processes, especially under dry conditions^42,43^. As ca. 50% of the sites have a mean annual WTDe of more than 30 cm, this may explain that a higher mean annual temperature predominantly led to a greater contribution of respiration-driven carbon losses at warmer sites.

Because XML models are fully data-driven, omitted factors (e.g., management) may have been attributed to predictors such as TA. The apparent peak of the TA effect near 9 °C (Fig. 3b) likely reflects clustering of managed sites in Denmark and northern Germany. Adding land-cover type as a predictor did not alleviate this peak (Fig. S5a, b). To address a temperature imbalance arising from grassland sites (only 11 of 75 grassland site-years have mean annual TA < 7.9 °C), we trained models on 10 resampled datasets, each time balancing land-cover types on both sides of a 7.9 °C split. Model responses remained consistent across these resampling iterations (Fig. S5c, d).

Finally, we looked more closely into the interaction effect of TA and WTDe on NBE. We determined the TA-WTDe interaction effect on a dataset with balanced land-cover types, equally representing site types both in the two TA regimes (above and below 7.9 °C) and along the range of WTDe (see “Methods”). We found an emergent interaction effect that became stronger with increasing WTDe both in the lower and the higher TA classes. At sites with warmer mean annual TA (>7.9 °C) the interaction term increased from near zero to a predominantly positive effect at WTDe deeper than 30 cm (Fig. S6). While there is substantial scatter in the interaction effect in the range of WTDe between 30 and 60 cm within the warmer TA class, the smoothed interaction and its confidence interval remained almost exclusively positive at WTDe of more than ca. 45 cm. This indicates that the combined effect of deeper WTDe and warm site conditions amplifies emissions beyond each individual effect. While to our knowledge, this pattern has not been reported on the annual and cross-site scale, it is consistent with evidence of an increased response of respiration to water table depth with higher temperatures^44^. Conversely, at cold sites (TA ≤ 7.9 °C) the smoothed interaction effect was predominantly negative at WTDe deeper than 30 cm, suggesting that the WTDe-driven emission response may be attenuated or partially suppressed at sites with colder temperatures (Fig. S6). This indicates that responses of annual peatland CO_2_ emissions to hydrology and climate are interconnected and potentially not uniform across climate regions, with implications for how emissions are extrapolated across peatlands differing in mean annual temperature.

However, it should be noted that the sample size of deeply drained grassland and cropland sites in the boreal region is very limited, with only seven site-years of TA < 7.9 °C and WTDe > 50 cm, resulting in a high uncertainty of the interaction effect at deep WTDe and low TA. (Fig. S6). To consolidate such general patterns and use them for temporal and spatial projections, prevailing gaps in the data need to be closed. Measurement networks such as the Dutch NOBV (Nationaal Onderzoeksprogramma Broeikasgassen Veenweiden) or the UK GHG Flux Network should be established in regions and land-cover types that are currently underrepresented. Particularly for deeply drained grassland and cropland sites in boreal regions, more observations are required to consolidate these findings. Future greenhouse-gas observation systems should prioritize identifying geographic and ecosystem-level gaps, establish targeted sampling in these hotspots of missing data and expand coordinated observation networks to ensure that the most policy-relevant and underrepresented peatland conditions are systematically monitored.

### Keeping water tables high buffers increasing emissions at high temperatures

**Fig. 4:**
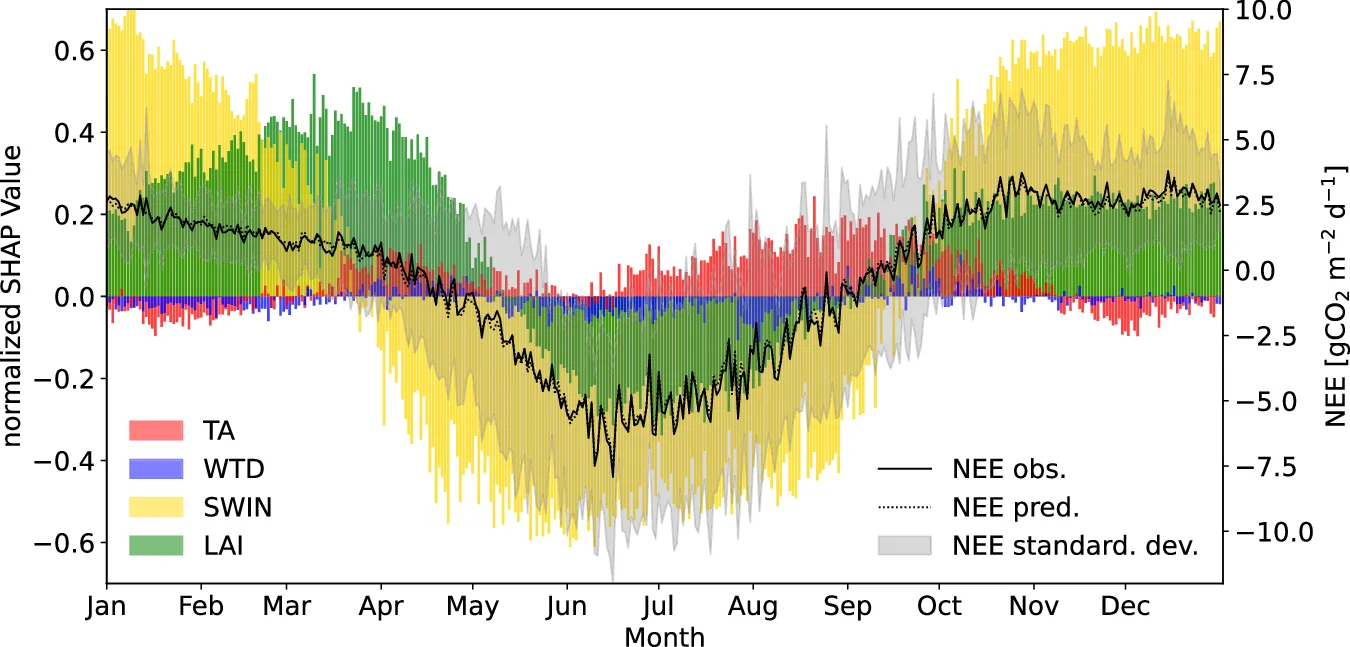
Seasonally dynamic effects of environmental controls on CO_2_ fluxes. The mean effects of the four drivers, temperature (TA), water table depth (WTD), short-wave incoming radiation (SWIN) and leaf area index (LAI) on daily CO_2_ fluxes (NEE) for each day of the year, averaged across 19 peatland sites. Standard deviations are calculated across all years and sites for each day. Effects are expressed as SHAP values derived from an XGBoost model. Positive values indicate an increase in CO_2_ emissions, negative values a reduction.

Data on annual CO_2_ fluxes provide valuable insights, but cannot capture seasonal and daily dynamics. To better integrate the effects of main predictors and their interactions, we analyzed 19 unmanaged peatlands with 113 site-years of daily eddy covariance data. Using the same XML approach, we trained a machine learning model across all site-years (*R*^2^ = 0.68 on test data of full years withheld during model training, Fig. S4). As there was no harvest on these sites and to be coherent with other studies, the CO_2_ flux is here called NEE (net ecosystem exchange). WTD is equivalent to WTDe at these sites, as peat depths are deeper than the deepest occurring WTD. The site-ID was a strong predictor of NEE, carrying 31% of the normalized variable importance. This highlights that site-specific aspects, such as vegetation cover or peat type, are crucial when modeling CO_2_ fluxes. (Fig. S4c, d). While the effect of WTDe dominated annually, SWIN and LAI were the strongest environmental predictors on the daily scale with 35% and 18% normalized importance. This reflects the slow daily changes of WTD versus the strong control of radiation and LAI on CO_2_ uptake. The effects of LAI and SWIN decreased NEE in summer, as expected (Figs. 4 and S7c, d). TA had little effect below ~14 °C, above which it increased emissions (Fig. S7a). The effect of WTD was similar to that on the annual scale, with small negative impacts at <20 cm, an emission increasing effect down to −60 cm, then plateauing (Fig. S7d).

We mapped the seasonal changes in driver effects across all sites (Fig. 4). LAI and radiation dominate CO_2_ fluxes, depicting the seasonal dynamics of photosynthesis. The effect of TA changes over the course of the year. Between mid-November and mid-February, low temperatures have little impact on CO_2_ fluxes. Between mid-February and the beginning of April, the effect of TA rises to an emission-increasing effect, likely depicting the impact of increasing temperatures on respiration before the onset of vegetation activity. Notably, between the start of April and the middle of June, this TA effect continuously reduces until it is nearly zero, around the same time the effect of LAI becomes most negative (thus uptake enhancing). Afterward, in mid- to late summer, the model attributed an emission-enhancing effect to higher temperatures (Fig. 4). This pattern aligns with a previous study, which found that warming induced increasing CO_2_ emissions in late summer and autumn and increased uptake in spring and early summer^45^. Towards the end of the hydrological year in September, when water tables are typically lowest, WTD exerts an emission-enhancing effect, coinciding with a strong emission-increasing effect of temperature to jointly amplify CO_2_ release.

**Fig. 5:**
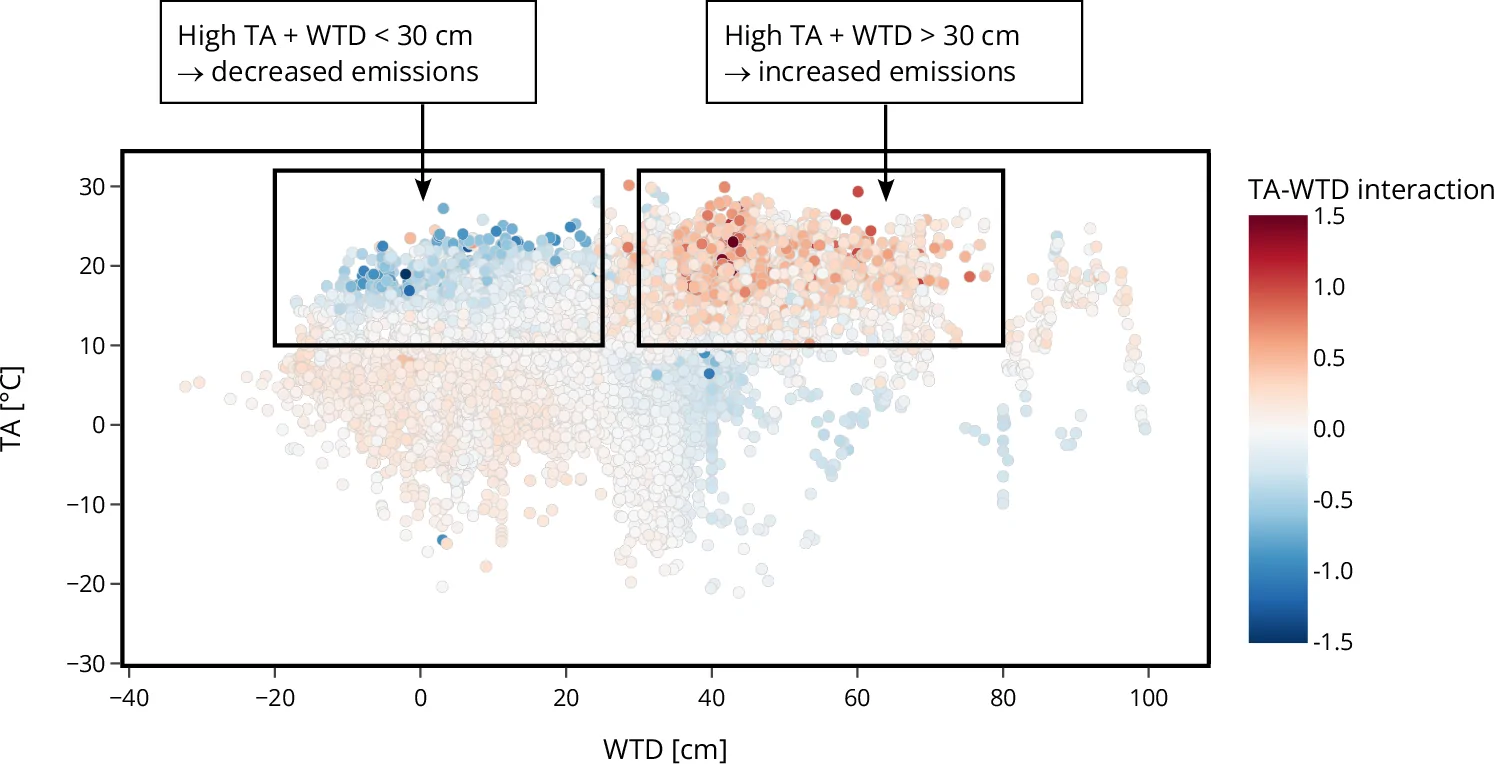
Deep water tables and high temperatures interact to increase CO_2_ emissions. The interacting effect of air temperature (TA) and water table depth (WTD) on daily CO_2_ fluxes across the range of temperatures and water tables in the dataset of 113 years of eddy covariance data from 19 peatland sites is determined with SHAP interaction effects. Interaction effects are a combined effect of TA and WTD that goes beyond the effect of each driver independently. A positive interaction effect (in red) denotes an emission-increasing effect while a negative effect (in blue) denotes a decreasing effect. At warm TA (above ca. 10 °C) and WTD of less than 30 cm, the negative interaction effect (in blue) denotes decreased emissions, while with deeper WTD the interaction effect becomes positive (in red), thus denotes increased emissions through combined high TA and deep WTD.

Using SHAP interaction values, we analyzed the combined effect of WTD and TA on daily CO_2_ fluxes (see “Methods”). On the daily scale, the divergent interaction between WTD and TA becomes even more apparent than on the annual scale (Fig. S8). With rising temperatures, the joint interaction becomes gradually stronger (Fig. 5). The effect of the interaction (increasing or decreasing emissions) varies along both TA and WTD gradients. At low temperatures and deep water tables, the interaction is close to zero or slightly negative, indicating lower emissions. At warm temperatures and shallow water tables (< 20 cm WTD), the interaction with high TA was negative. In contrast, deeper WTD interacted with high TA to further increase emissions. This interaction was a consistent result found across 113 years of in-situ observations from multiple peatland types, previously detected only at the site level^46,47^ and peat column experiments^19,48^. It implies that maintaining water tables close to the surface not only directly reduces emissions through a smaller volume of aerated peat but may also buffer the impact of rising air temperatures on CO_2_ emissions.

## Methods

### Annual CO_2_ flux data and selection of study sites

We present net ecosystem CO_2_ uptake as net biome exchange (NBE). This is the balance of all CO_2_ released through respiration and taken up via photosynthesis, additionally including harvest as a loss, and deposition of organic material through fertilizer as an input^24^. We do not use the term net ecosystem carbon balance (NECB) here because NECB typically includes other carbon fluxes such as dissolved organic carbon or methane^49^. To compile the largest existing collection of annual CO_2_ budgets, we searched the literature and included data from sites across Europe and North America, measured with chambers (manual or automatic) or with the eddy covariance method. We included sites characterized in the literature as peatlands with direct annual CO_2_ flux budgets and water table depth measurements. Sites without peat-forming vegetation, and those that are heavily degraded (e.g., managed grasslands and croplands), were included, provided they are classified as peatland in the source literature and situated on organic peat soils with high organic carbon content. Sites with more than a marginal occurrence of trees were excluded. For six sites, only reports of growing season water table depths were available, all of which are boreal sites in which the ground is likely frozen through most of the non-growing season. For these sites (Stordalen, Lompoljankka, Fajemyr, Athabasca, Attawapiskat, Kaamanen, Kinoje), we used the mean growing season water table depth.

In some northern ecosystems, chamber measurements were restricted to the growing season. For these sites, if non-growing season data were unavailable, we selected sites that report growing season respiration and used the IPCC wetlands supplement methodology, which assigns 15% of growing-season CO_2_ respiration emissions to the non-growing season^50^. For sites with active biomass harvesting, only sites were included that had measurements of exported biomass, which was then incorporated into the on-site CO_2_ flux measurements to generate NBE estimates. For deeply drained sites where the water table depth could potentially go lower than the depth of the peat column, sites were included when peat depth measurements were available, allowing for the calculation of effective water table depth as the depth of the aerated peat column.

This resulted in a total of 114 sites and a total of 276 site-year annual NBE budgets (Fig. 1 and Supplementary Data). Study sites were broadly classified based on their land-cover type and adhering to classifications in the source literature. Grasslands are sites that are drained for grass cultivation, mostly used as pastures or for biomass production. Croplands are drained sites with active agricultural crop cover. Within the unmanaged sites, we adhered to the typical classification generally dividing peatlands into fens, with tendentially higher nutrient availability, which often receive groundwater from the surrounding watershed, and typically rainwater-fed and nutrient-poor bogs.

### Compilation of half-hourly CO_2_ fluxes

To assess the main drivers of CO_2_ fluxes in different peatland types, high-resolution observations are needed. Thus, from the aggregated list of sites, we collected eddy covariance flux measurements from sites with retrievable half-hourly or daily data, yielding the longest time series of daily EC observations across available peatland sites. Data for FR-Lgt, UK-Amo, FI-Sii and SE-Deg were acquired from the ETC L2 Fluxes and Meteo products from the ICOS Carbon portal^51–58^. Data for NL-Hor and older data for UK-Amo were extracted from the Warm Winter 2020 ecosystem eddy covariance flux product^59^. Data for CA-Mer was extracted from AmeriFlux^60^. Daily fluxes for UK-Cro and UK-Bak, as well as half-hourly fluxes for the site UK-Aln, were retrieved via the NERC Environmental Information Data Center^61,62^. All other EC flux data were extracted from the GHG-Europe database or provided by site PIs directly. This led to a database of 19 peatland sites distributed across nine countries in Europe and Canada (Table S1 and Fig. 1). Only unmanaged sites were included for the analysis of daily CO_2_ fluxes. Biomass removal during harvest alters the CO_2_ flux dynamics in a form that is not attributable in a time-series model, as they are singular events unrelated to the predictors included in the model. Additionally, only site-years with concurrent CO_2_ flux, TA, SWIN and WTD measurements were included, leading to a dataset spanning a total of 113 site-years. The data was provided in different states of completeness, spanning from unfiltered half-hourly flux data to gap-filled and processed data. Therefore, a standardized approach was taken to post-process all time series to span the full range of available data, including extra steps of quality filtering and gap filling (see details in the following section). Wherever data was already published before, we adhered as closely as possible to the filtering processes described in the respective literature or by directly consulting the PIs (e.g., filtering out data during rain events or at low signal strengths when open-path gas analyzers are used).

### Auxiliary environmental data

We assessed the relationship between daily or annual CO_2_ fluxes with environmental variables using observations of air temperature, solar radiation, groundwater table, and leaf area index.

For the dataset of annual budgets, annual radiation is rarely provided in the respective literature; daily incoming radiation was extracted from the ERA5 Land dataset^63^ (9 km resolution) and aggregated to the annual scale. LAI was extracted from the 4-daily MODIS leaf area index product MCD15A3H^64^. Satellite-derived data, such as MODIS LAI, can have gaps when sensors fail or when cloudy weather is encountered. This can create imbalances in the datasets, e.g., when specific periods are repeatedly missing. We computed monthly means from the 4-daily LAI for each site and interpolated these using a Savitzky-Golay filter^65^ using the “SciPy”^66^ package in Python to achieve a full monthly time series. These filled and smoothed LAI time series were used to compute the annual mean LAI for each site-year. For the daily timescale, the monthly means were first linearly interpolated and then smoothed with a moving mean on a 7-day running window.

Water table data for NL-Hor was extracted from the input files used in a modeling study^67^. Water table data for UK-Aln, UK-Bak and UK-Cro were extracted from literature^68^ using the *Automeris WebPlotDigitizer* v. 4.5^69^.

### Data processing

Apart from sites with EC measurements, annual budgets were directly extracted from the literature; no further processing was conducted on them. For the sites with high-resolution EC measurements, annual sums were calculated after post-processing and gap-filling fluxes as described below.

Post-processing of the daily CO_2_ fluxes depended on the source from which the data were acquired. All data was received as aggregated half-hourly timesteps, but in some cases, additional post-processing, such as filtering and gap-filling, was required to achieve a reliable, high-quality time series of daily data. A pipeline was implemented to achieve harmonized and comparable data (Fig. S9).

Data acquired from ICOS or Ameriflux databases are already processed with a standardized approach for filtering out non-turbulent conditions and outlier removal^70^. Flux data provided directly from PIs or via GHG-Europe were available in miscellaneous formats and varying states of completeness. Data from GHG-Europe is not actively processed by the network anymore, and level 2 data obtained from this database only goes through a rough pre-filtering. For data that was unfiltered and showed large amounts of noise and spikes, we applied several steps of filtering. First, absolute limits were applied when extreme spikes could easily be identified, basing the limits on the 99th percentile of the highest quality flux data. If the data remained noisy and still showed spikes, a despiking was conducted using a moving window of 14 days and removing fluxes with an offset of two standard deviations from the window mean. Poorly developed turbulent conditions were removed using u*-filtering with REddyProc^71^. Additional filters such as for precipitation events (when an open-path analyzer was used), turbulence characteristics, or outlier removal were applied as described in the respective literature for some sites^72–75^. Finally, CO_2_ fluxes and relevant predictors were visually checked for erratic periods to remove unrealistically high or low values or fluctuations.

To create the daily time series and annual budgets from half-hourly data, the resulting gaps in the NBE time series had to be filled. The standard gap-filling procedure (used in ICOS or Ameriflux) using marginal-distribution sampling (MDS) has been shown to be outperformed by machine-learning methods with increasing lengths of data-gaps^76^ especially in northern ecosystems^77^. To ensure a comparable gap-filling quality across our dataset, we removed CO_2_ fluxes filled with MDS and applied the XGBoost gap-filling method to all sites. However, to fill gaps in the flux data, required to first fill gaps in the ancillary data. For variables that are well-predictable based on meteorological observation data (e.g., TA, SWIN, etc.), we applied XGBoost regression tree models^78^ with meteorological variables from the same dataset that are closely correlated to the target variable as predictors. We tested performances for these models on data not included in model training. Average *R*^2^ and its confidence interval across sites and meteorological variables was 92 ± 2%. To fill water table data, we constructed 30-day rolling sums of precipitation to estimate a water availability index, which was then used as a main predictor along with soil temperature, soil water content and precipitation amounts to fill water table time series. The average variance explained of the models filling the water table was 91% ± 5%. Finally, CO_2_ fluxes were gap-filled using XGBoost and the filled ancillary data. The average *R*^2^ of the CO_2_ gapfilling across sites was 82 ± 5%. With this filled dataset, we calculated a time series of daily mean NBE (in µmol m^−2^ s^−1^) for each site.

### Linear and non-linear regression analysis

To test the relationship between annual CO_2_ budgets and effective water table depth, we used an ordinary and a multiple linear regression model, a Gompertz model and a LOESS (locally estimated scatterplot smoothing) function with a span of 0.6. The non-linear Gompertz function was fitted using the “minpack.lm” package in R^79^, the ordinary and multiple linear models, as well as the LOESS fits, were created with the “stats.lm” packages included with the R core packages. We assessed model assumptions of the linear model using residual diagnostics as implemented in the “DHARMa” package^80^. We evaluated overall residual structure using a Kolmogorov–Smirnov uniformity test and inspected potential heteroscedasticity by examining quantile-based residual patterns across fitted values. For the LOESS and Gompertz models, tests for heteroscedasticity were conducted with a Breusch-Pagan test.

The Gompertz function was calculated as in the approach outlined in previous studies^12,13^:

1 $${{{\rm{NBE}}}}={{{\rm{NB}}}}{{{{\rm{E}}}}}_{\min }+{{{\rm{NB}}}}{{{{\rm{E}}}}}_{{{{\rm{diff}}}}}*{e}^{-a*{e}^{b*{{{\rm{WTDe}}}}}}$$

where NBE_min_ refers to the lower asymptote, NBE_diff_ is the span between the lower and upper asymptote, *a* is the displacement along the WTDe axis and *b* defines the gradient. Extraction of the parameters and calculation of the variance-covariance matrix to calculate standard errors and confidence intervals for the Gompertz function was done using the “stats” package in R. To calculate confidence intervals for the Gompertz-function, we constructed the Jacobian matrix of partial derivatives of the function with respect to each model parameter. The uncertainty in the predictions was then estimated by propagating the joint uncertainties in the model parameters through the prediction function. This was achieved by performing a matrix multiplication of the Jacobian matrix with the variance-covariance matrix of the parameter estimates. The resulting matrix provides the variance of the predictions, from which the standard errors and confidence intervals were derived.

A LOESS function is essentially a classical least-squares fit, but it allows for increased flexibility for the cost of increased computation. The model fits locally along the range of the predictor, including a tunable portion of the surrounding data in the fitting process. As the model assumes a smooth function, a weighting function is applied that assigns higher weights to data close to each evaluated point^27^.

The residuals of the models were tested for heteroscedasticity with the Breusch-Pagan test. We further tested models on signed log-transformed NBE to mitigate heteroscedasticity by symmetrically compressing positive and negative extremes while preserving flux direction.

Mixed linear effects models were constructed in R using the “lme4*”* package. Conditional and marginal *R*^2^ values for the mixed effects models were calculated using the “MuMIn*”* package^81^.

### Driver analysis using explainable machine learning (XML)

To understand what drives NBE, we chose the XGBoost implementation in Python and R^78^ for its high degree of tunability and good performance previously demonstrated on gas flux data^77,82,83^. To avoid including collinear driving variables, which may obscure the true relationship between predictors and their effects, a careful selection was undertaken to construct the models. We included TA, SWIN, WTD satellite-based LAI (see above) and the site ID as predictors. For the daily data, the time series of LAI was derived with the same procedure as for the annual data using a Savitzky-Golay filter. The site ID was included as a predictor to enable the models to account for site-specific differences not attributable to the drivers in the model, such as differences in soil properties, vegetation, microtopography or management. These categorical variables were encoded as one new predictor for each site, which has a value of zero or one to indicate site-type (Dummy-Encoding).

For the model on the annual data, we trained models on all available site-years, iteratively leaving out all data from 5 randomly selected sites until all sites had been left out once, providing a variety of leave-one-out cross-validation (LOOCV). The final reported root mean square error (RMSE) and *R*^2^ (calculated equivalent to Nash-Sutcliffe efficiency) are calculated as averages across all training rounds. SHAP values and SHAP interaction values were extracted from the models directly from the “XGBoost” package^78^. To determine an optimal split point in the annual data with respect to the response to TA, we trained an XGBoost regression model restricted to a tree depth of 1 (a single-split decision stump) with the SHAP value for TA as the predictor and TA as the predictand. We then extracted the split value from the regression tree. We tested whether the SHAP-derived contribution of TA to modeled NBE differed above and below the threshold using a Wilcoxon rank-sum test. This value represents the threshold that best partitions the data to minimize the prediction error (equivalently, maximizes the reduction in response variance) across all sites. Finally, we derived the TA-WTDe interaction effects in both temperature regimes. Since the combined effects of WTDe and TA may be influenced by an imbalance in land-cover types, both along the TA and WTDe axes, we resampled the data such that in every bin of 20 cm WTDe, both TA regimes had the same number of sites per land-cover category. Because this resampling is subject to the randomness of the sampled site, this procedure was repeated 50 times, each time sampling the data, training models and extracting the SHAP interaction for the TA-WTDe interaction (Fig. S10). The resulting figure (Fig. S6) shows the average response function as well as the confidence interval among the fits on the 50 resampled datasets.

For the analysis of daily data, those days when more than 50% of the data was gap-filled were excluded to avoid potential bias in the model structure. As the models used for gap-filling had more predictors than the models used for the driver analysis, and the gap-filling was conducted on a half-hourly scale while the driver analysis was done on the daily scale, we deem this filtering sufficient to decouple the models. We trained one model using the same predictors as for the annual dataset across all sites. Hyperparameters were tuned using a 10-fold grid search cross-validation. We assessed model performance using blocked cross-validation, holding out three entire years (across all sites) as test sets with a 7-day embargo period around each held-out year to remove residual autocorrelation at block boundaries. The size of the validation datasets varied from 6–15% of the total data, depending on the sampled years. The reported RMSE and *R*^2^ are the means across all cross-validation folds. Site identity was retained as a categorical predictor throughout; the reported validation metrics reflect generalization across time, not to unobserved sites. The final model reported here was refit on the complete dataset using the hyperparameters validated via blocked cross-validation, and SHAP values were derived from this full-data model. For the XML analysis, we used Shapley Additive Explanations (SHAP values)^84^. Specifically, we used the implementation of “TreeExplainer” included in the Python “shap” package^85,86^ for the daily analysis and its integration in the R package “XGBoost” for the annual analysis. The method is rooted in game theory and calculates the marginal contribution of each predictor variable to each predicted datapoint. The SHAP value is a measure of how much the effect of the specific variable changes the prediction from a base value. This base value is, by default, the mean prediction of the model. This method allowed us not only to evaluate overall variable importances but also to dynamically assess how the effect sizes change over time. A SHAP value shows how the mean value of the response variable changes due to each specific driver at each timestep. A positive value denotes an increasing effect, a negative value a decreasing effect. In our case, a positive SHAP value denotes a marginal effect that increases NBE, thus increasing net CO_2_ emission and vice versa. Similar approaches have successfully been deployed to determine drivers of respiration and drought effects in forests^87–89^ or governing variables of regional Dutch peatland fluxes^17^. For the daily scale analysis, we derived the SHAP values from the final cross-validated model. For the annual scale, the regression trees are much more variable due to the smaller sample size. We therefore trained 30 models and derived SHAP values each time, finally using the mean SHAP value across all models for the dependence plots and the mean and standard deviations across all models to show mean absolute importances.

## Supplementary information

Supplementary Information

Description of Additional Supplementary File

Supplementary Dataset 1

Transparent Peer Review file

## Data Availability

Daily CO_2_ fluxes used for the XML analysis and the 276 annual CO_2_ budgets generated in this study have been deposited in the Codeocean capsule attached to this article, available under the accession code https://doi.org/10.24433/CO.1911864.v1.

## Code availability

All code necessary to reproduce the figures, models and analysis is available in the Codeocean capsule attached to this article under the accession code https://doi.org/10.24433/CO.1911864.v1.
