# Supplementary material for: Drivers of northern peatland CO2 fluxes revisited: interacting water level-temperature dependency
Source: Nat Commun. 2026 Sep 4;17:9504. doi: 10.1038/s41467-026-77456-6 (PMC13545207; doi:10.1038/s41467-026-77456-6)
Supplement: Supplementary file 1 [file 41467_2026_77456_MOESM1_ESM.pdf]

Supplemetary for: Drivers of northern peatland CO_2 fluxes revisited: interacting water level-temperature dependency

Table S.1 Table of peatland sites used for the explainable machine learning analysis of drivers of daily CO_2 fluxes. Site-IDs contain a country code (before the dash) and a station code (after the dash). Climate conditions are categorized according to the Koeppen-Geiger classification. Avg. TA is the mean annual temperature, avg. P the mean annual precipitation, avg. WTD the mean annual water table depth. The reference relates to the literature containing site information.

Site ID	Climate	Lon.	Lat.	Type*	Avg. TA [°C]	Avg. P [mm]	Avg. WTD [cm]	Years used in model	Ref. (site infos)
CA-Bdb	Dfb	-69.43	47.58	Bog	8.6	954	29.4	4	41
CA-Db2	Csb	-123.00	49.12	Bog	10.7	1128	2.4	4	42,43
CA-Dbb	Csb	-122.99	49.13	Bog	12.2	1128	2	8	44,45
CA-Mer	Dfb	-75.52	45.41	Bog	7.3	585.5	37.2	23	46
CA-Sak	Cfb	-69.61	47.73	Extraction	7.1	954	45.4	2	47
DE-Amv	Dfb	6.96	52.18	Bog	11.3	748	26.4	1	48
DE-Bou	Dfb	7.18	52.65	Bog	11.2	813	11.5	3	49
DE-Utm	Dfb	8.83	52.51	Bog	10.4	673	-7	1	50
EE-Pts	Cfb	24.37	58.56	Extraction	8.4	870	80	1	-
FI-Sii	Cfb	24.19	61.83	Fen	6.8	711	5.9	8	51
FR-Frn	Cfb	6.17	46.83	Fen	6.8	1020	12.3	4	52,53
FR-Lgt	Cfb	2.28	47.32	Fen	13	699	26.1	4	54
IE-Kil	Cfb	-9.90	51.97	Bog	10.7	1460	4.2	9	47
NL-Hor	Cfb	5.08	52.24	Fen	11	800	24.4	7	55
SE-Deg	Cfc	19.56	64.18	Fen	7.5	523	7	6	56
UK-Aln	Cfb	-4.30	53.31	Fen	9.6	887	10.9	1	22
UK-Amo	Cfc	-3.24	55.79	Bog	9.1	960	18.1	18	57
UK-Bak	Cfb	0.29	52.31	Fen	10.2	640	25.6	3	32
UK-Cro	Cfb	-3.96	58.37	Bog	7.3	949	7.7	6	58

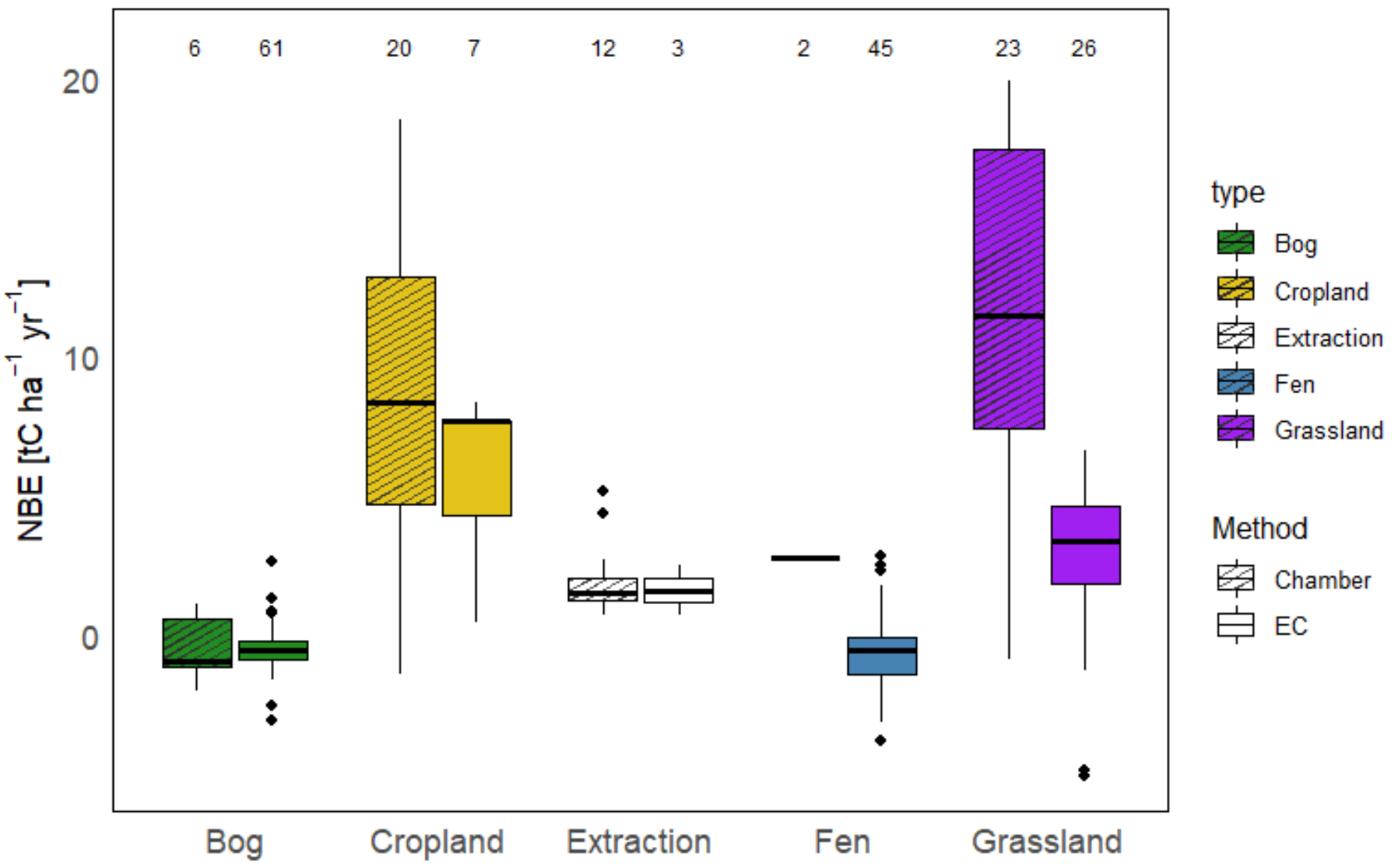

Figure S.1 Annual sums of net biome exchange (NBE) stratified by site land-cover type and CO_2 flux measurement methods. Numbers above each box denote the number of site-years for this combination of land-cover type and method. Horizontal lines within the boxes show the median value, the box stretches from the 25th (Q1) to the 75th (Q3) percentile. Whiskers stretch to 1.5 times the interquartile range (IQR) beyond Q1 and Q3, with points beyond the whiskers representing potential outliers.

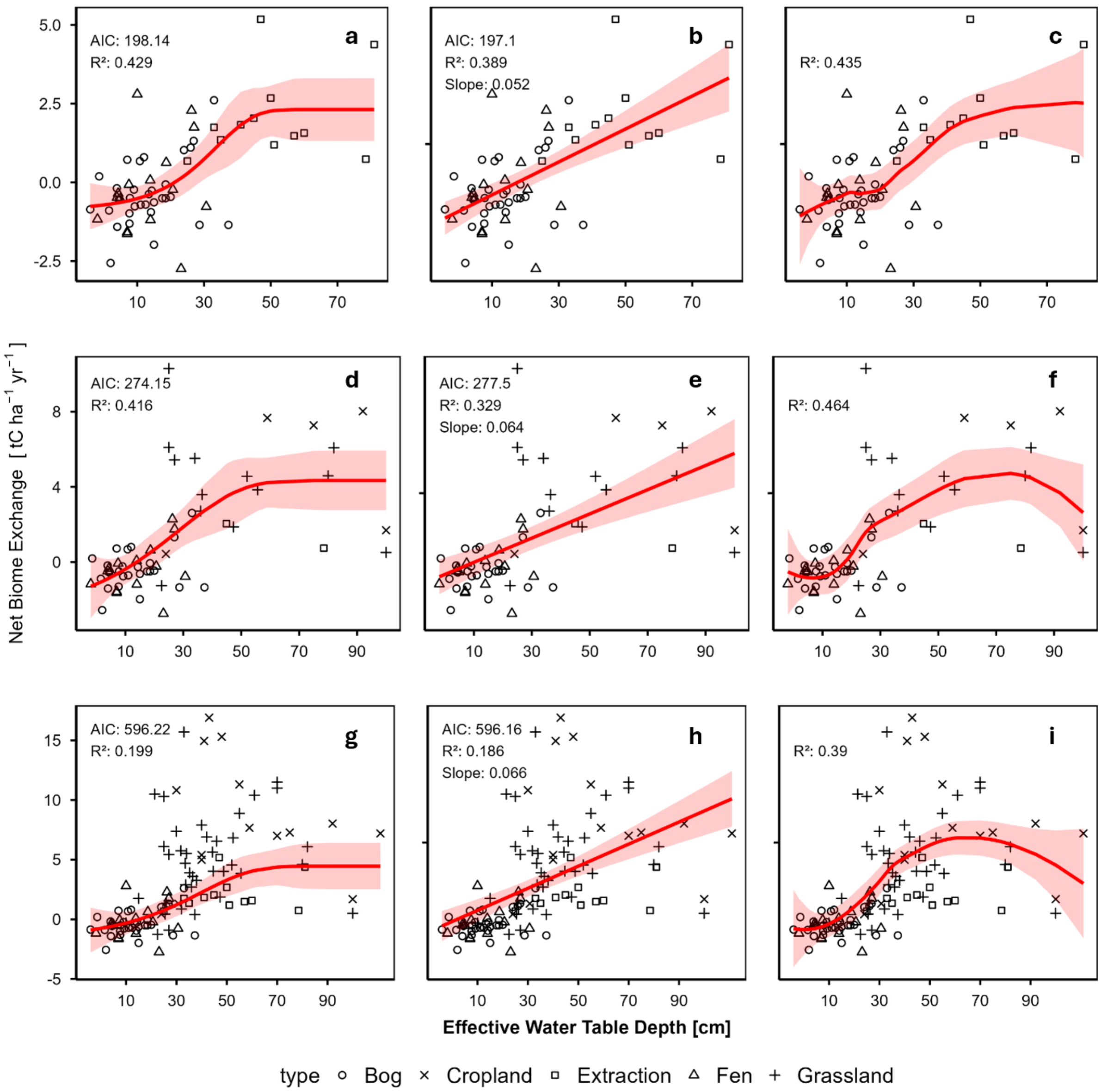

Figure S.2 Nonlinear models fit the relationship between effective water table depth and net biome exchange better than a linear one on multiple subsets of data. Gompertz (**a, d, g**), linear (**b, e, h**) and LOESS (**c, f, i**) fits for effective water table depths against net biome exchange (NBE) excluding managed sites (**a, b, c**), against NBE derived exclusively by Eddy Covariance (**d, e, f**) and against net ecosystem exchange (NEE), which does not include harvest(**g, h, i**).

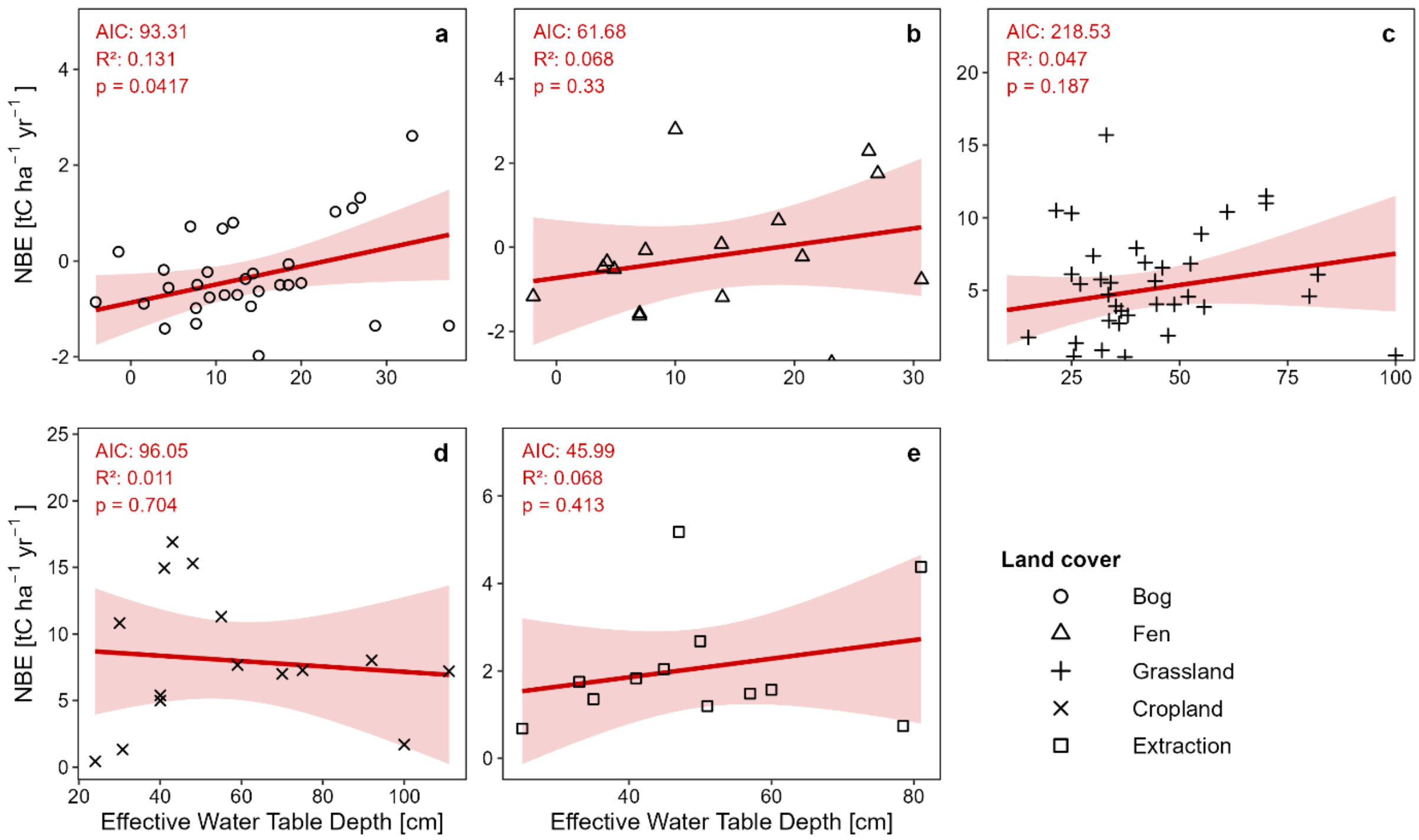

Figure S.3 Linear models for net biome exchange (NBE) against effective water table depth (WTDe) separated by land-cover types are not significant. One linear model was fit for the WTDe-NBE relationship for the land-cover types bog (**a**), fen (**b**), grassland (**c**), cropland (**d**) and extraction sites (**e**).

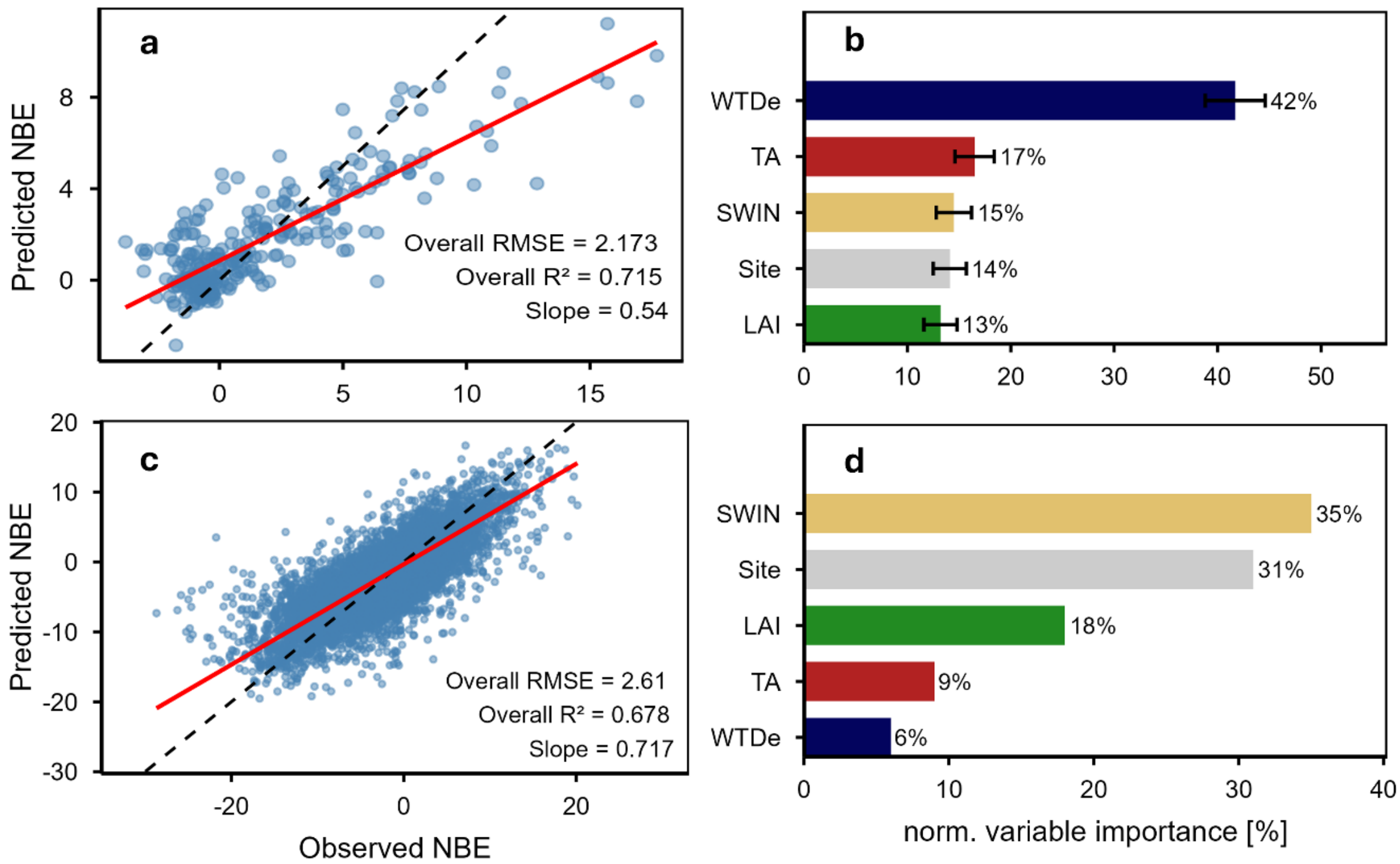

Figure S.4 Model performance metrics and driver importances of the models used for explainable machine learning. The model performances are derived on independent test datasets and driver ranks are based on mean absolute SHAP values for the XGBoost models predicting annual (**a, b**) and daily (**c,d**) net biome exchange. Shown drivers are solar radiation (SWIN), leaf area index (LAI), air temperature (TA) and effective water table depth (WTDe).

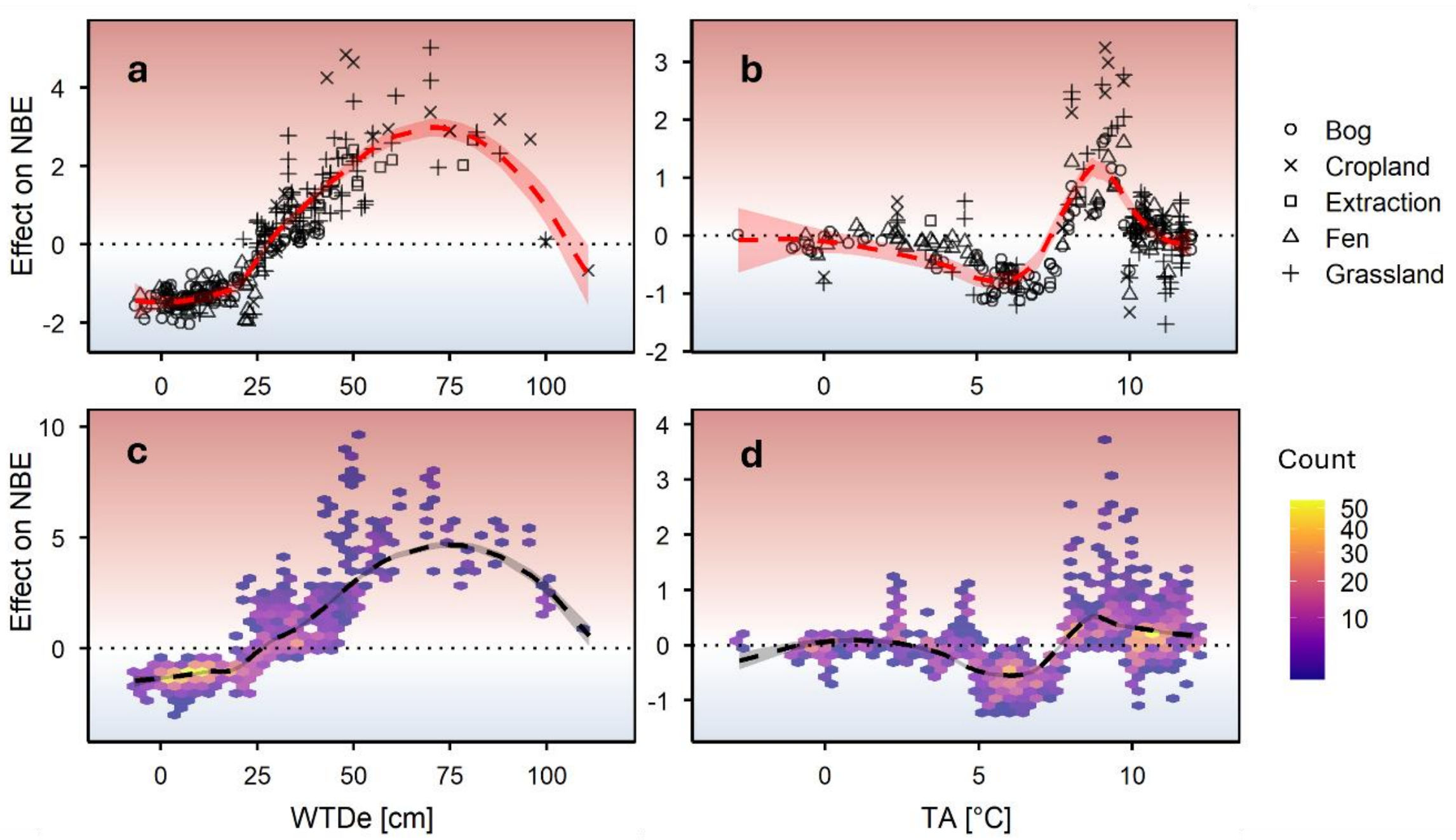

Figure S.5. Response shapes based on explainable machine learning remain stable when adding predictors or balancing the dataset across warmer and colder sites. Effect of effective water table depth (WTDe) and air temperature (TA) on net biome exchange (NBE) based on XGBoost models with (**a, b**) categorical variables for the land-cover types included as predictors and (**c, d**) derived from 10 iteratively resampled datasets in which for all land-cover types the same number of sites is included in the data above and below 7.9 °C.

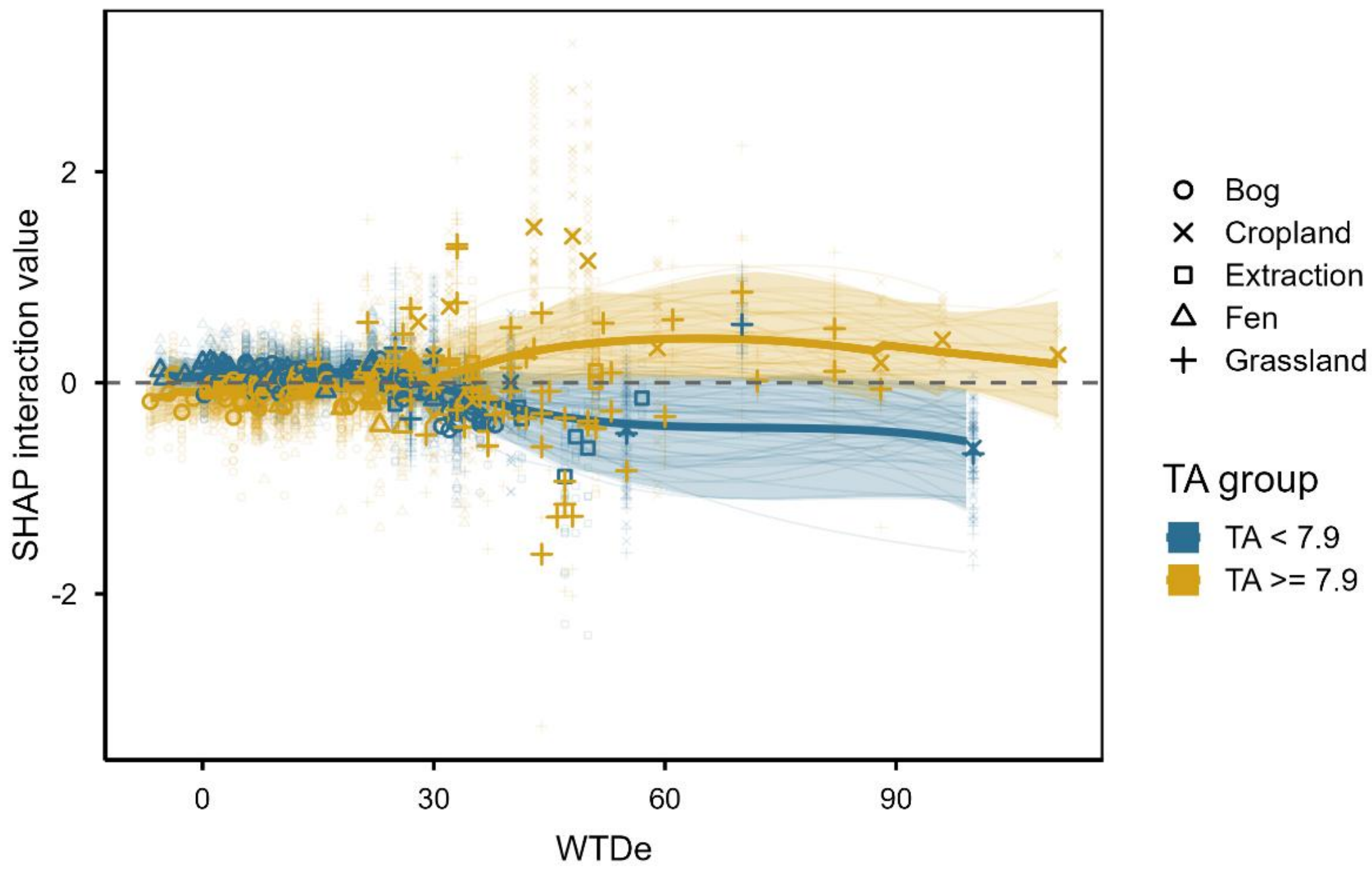

Figure S.6 SHAP interaction effects of effective water table depth (WTDe) and air temperature (TA) enhance emissions at lower WTDe at warm sites. Two LOESS-fits are done on data with mean annual air temperatures above (orange) and below (blue) 7.9 °C. The dataset was resampled 50 times, each time ensuring that the datapoints per land-cover type above and below 7.9 °C as well as within every 20 cm bin of WTDe are equal. Faint symbols and lines show the resampled datapoints and loess fits and the thick symbols and lines the mean interaction effect across all resamplings the datapoint was included in and mean LOESS fits.

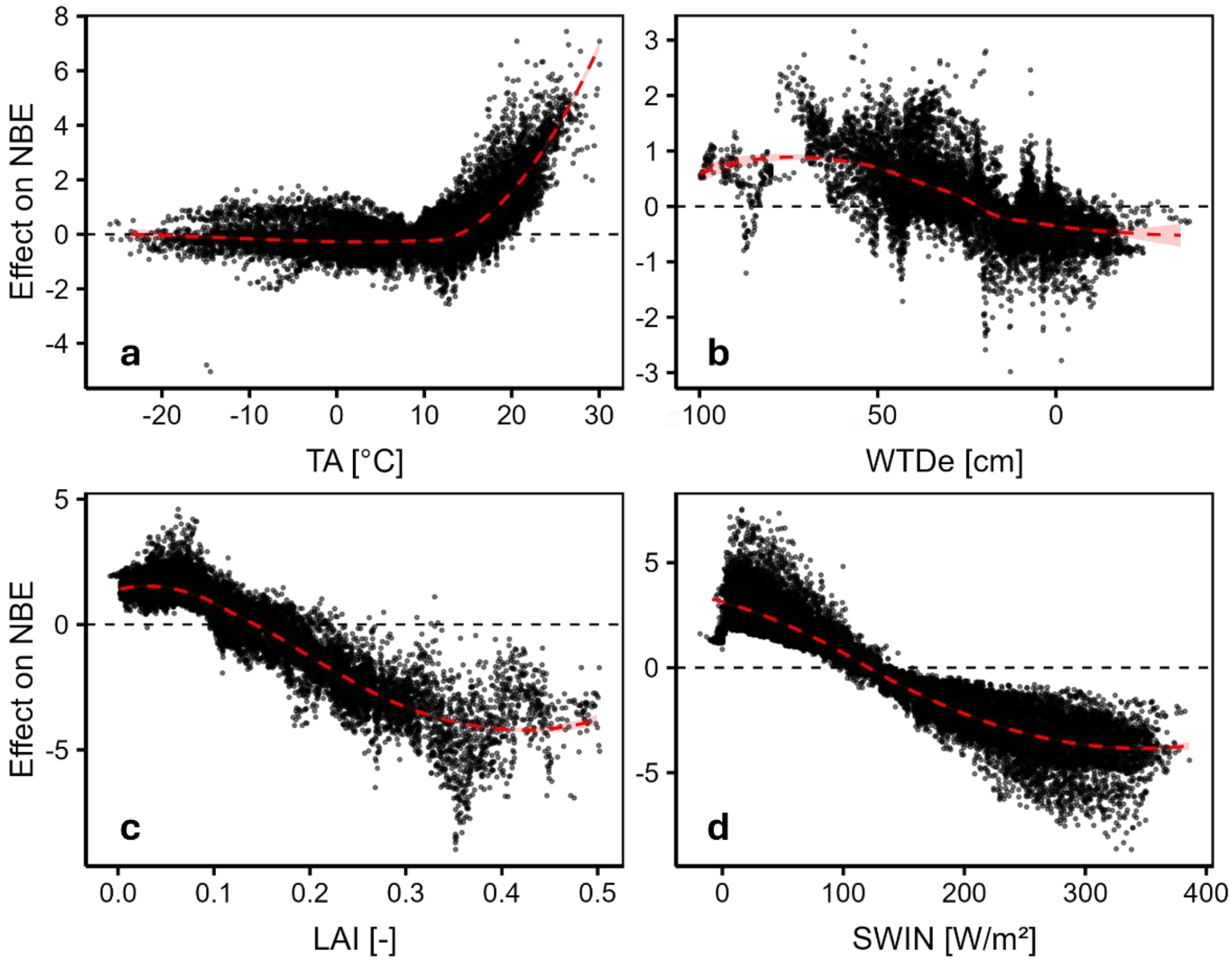

Figure S.7 Dependency of net biome exchange on the daily scale on the four environmental predictors. The points denote the effect of the driver air temperature (TA) (**a**), effective water table depth (WTDe) (**b**), leaf area index (LAI) (**c**) or short wave solar radiation (SWIN) (**d**) on net biome exchange (NBE) under the respective conditions. Red lines depict a LOESS fit, showing a smoothed response curve for each driver.

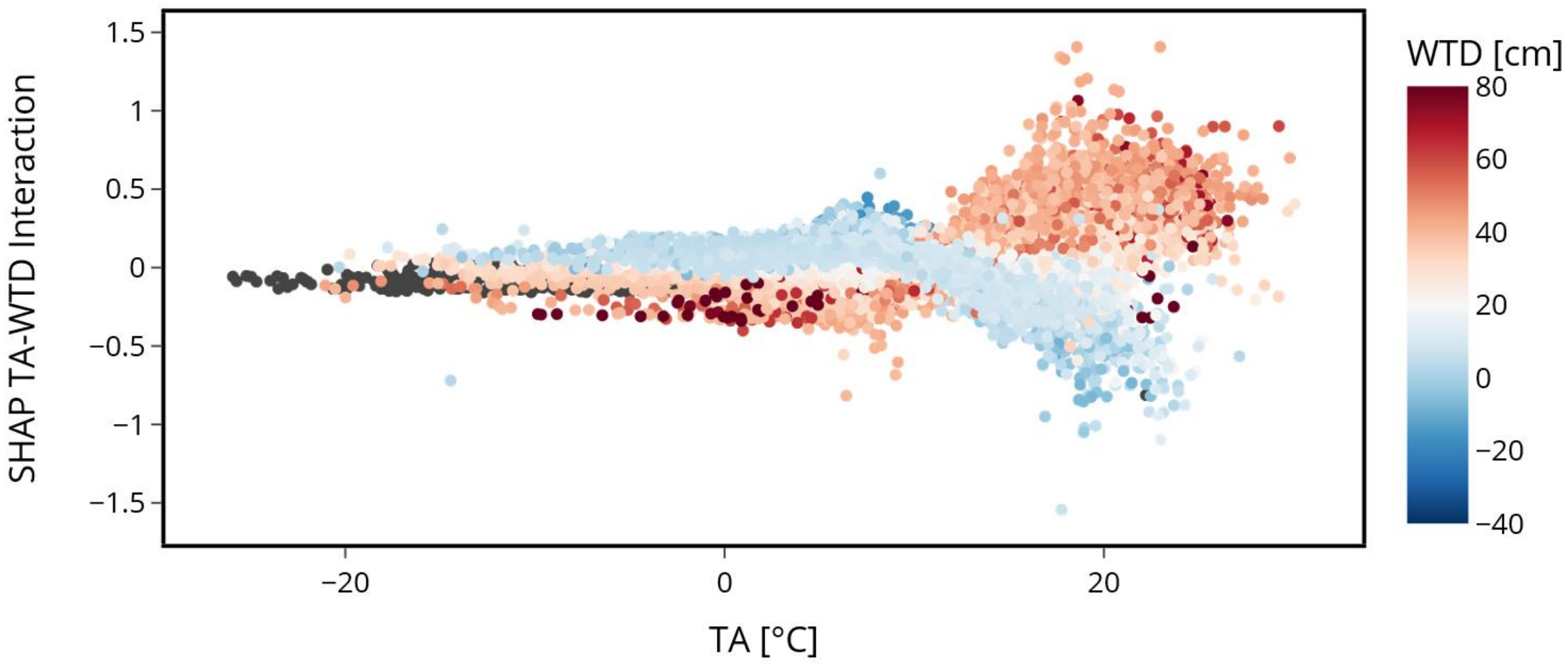

Figure S.8. SHAP interaction values of air temperature and water table depth for the model trained on daily net biome exchange across 19 peatland sites. SHAP Interaction values describe the combined effect of air temperature (TA) and water table depth (WTD) beyond the effect each driver exerts alone. At high WTD the effect of TA becomes predominantly negative at high TA, thus decreases emissions, while at a high WTD the effect of TA becomes positive, favoring emissions.

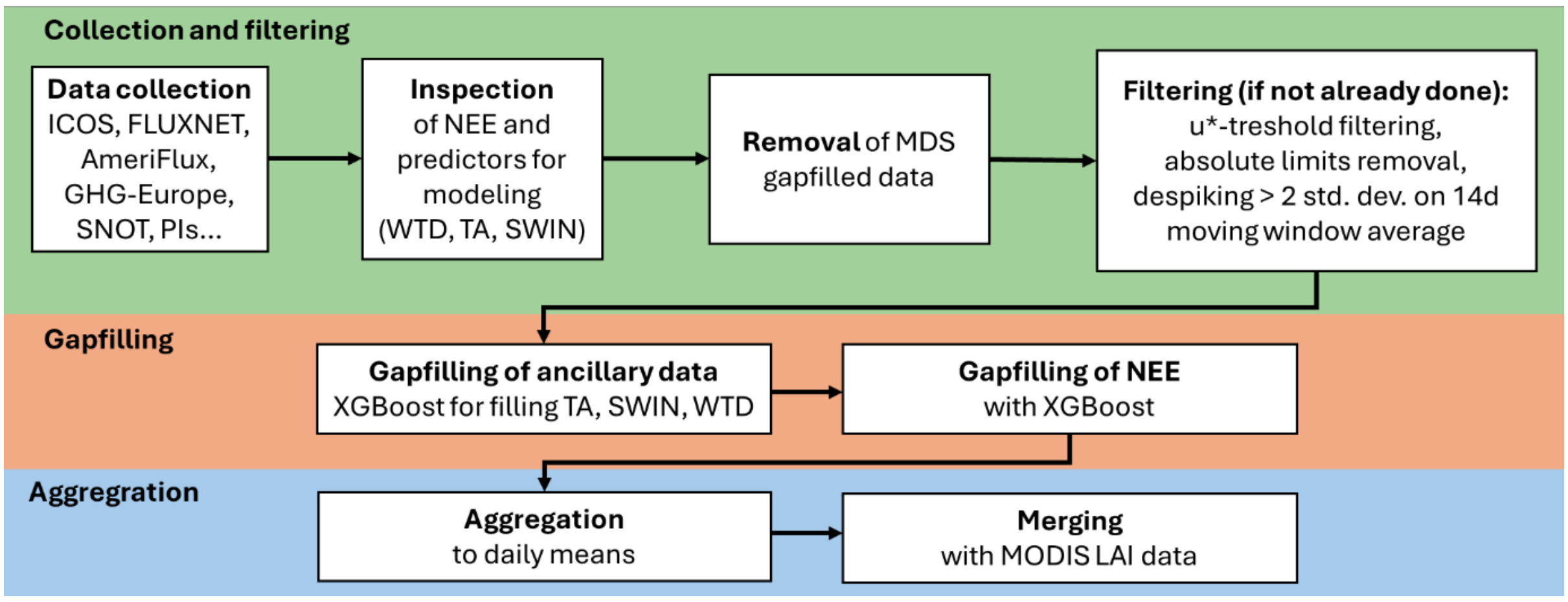

Figure S.9: Pipeline used to construct the dataset of daily CO_2 fluxes for the sites used for the daily driver analysis. Flowchart illustrating the pipeline used to compile and process the peatland CO_2 flux data used for the driver analysis.

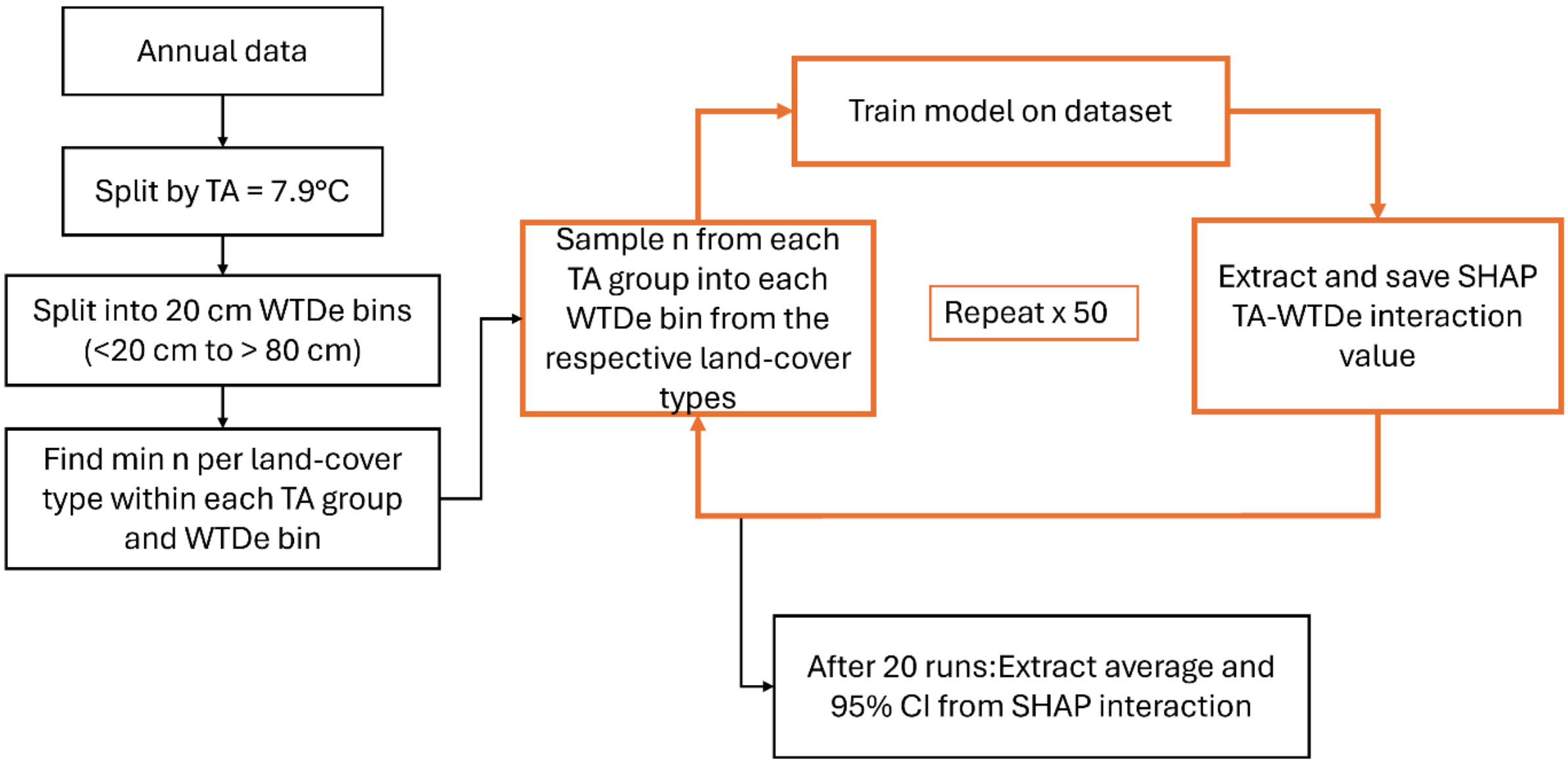

Figure S.10: Illustration of the iterative resampling procedure. The flowchart illustrates the resampling pipeline to produce the SHAP interaction values of air temperature (TA) and effective water table depth (WTDe) on data with balanced land-cover types across the TA split of 7.9 °C and across all WTDe bins.

Supplementary References

1. Drollinger, S., Maier, A. & Glatzel, S. Interannual and seasonal variability in carbon dioxide and methane fluxes of a pine peat bog in the Eastern Alps, Austria. Agric. For. Meteorol. 275, 69–78 (2019).

2. Humphreys, E. R., Charron, C., Brown, M. & Jones, R. Two Bogs in the Canadian Hudson Bay Lowlands and a Temperate Bog Reveal Similar Annual Net Ecosystem

Exchange of CO_2. Arct. Antarct. Alp. Res. 46, 103–113 (2014).

3. Strachan, I. B., Pelletier, L. & Bonneville, M.-C. Inter-annual variability in water table depth controls net ecosystem carbon dioxide exchange in a boreal bog. Biogeochemistry 127, 99–111 (2016).
4. Wang, M. et al. Can abandoned peatland pasture sequestrate more carbon dioxide from the atmosphere than an adjacent pristine bog in Newfoundland, Canada? Agric. For. Meteorol. 248, 91–108 (2018).
5. Helbig, M. et al. Direct and indirect climate change effects on carbon dioxide fluxes in a thawing boreal forest–wetland landscape. Glob. Change Biol. 23, 3231–3248 (2017).
6. Helbig, M., Quinton, W. L. & Sonnentag, O. Warmer spring conditions increase annual methane emissions from a boreal peat landscape with sporadic permafrost. Environ. Res. Lett. 12, 115009 (2017).
7. Beetz, S. et al. Effects of land use intensity on the full greenhouse gas balance in an Atlantic peat bog. Biogeosciences 10, 1067–1082 (2013).
8. Beyer, C. & Höper, H. Greenhouse gas exchange of rewetted bog peat extraction sites and a Sphagnum cultivation site in northwest Germany. Biogeosciences 12, 2101–2117 (2015).
9. Järveoja, J. et al. Impact of water table level on annual carbon and greenhouse gas balances of a restored peat extraction area. Biogeosciences 13, 2637–2651 (2016).
10. Dyukarev, E. A. et al. Net Ecosystem Exchange, Gross Primary Production And Ecosystem Respiration In Ridge-Hollow Complex At Mukhrino Bog. Geogr. Environ. Sustain. 12, 227–244 (2019).
11. Lund, M., Lindroth, A., Christensen, T. R. & SträöM, L. Annual CO_2 balance of a temperate bog. Tellus B Chem. Phys. Meteorol. 59, 804–811 (2007).
12. Olefeldt, D. et al. Net carbon accumulation of a high-latitude permafrost palsa mire similar to permafrost-free peatlands. Geophys. Res. Lett. 39, (2012).
13. Evans, C. D. et al. Overriding water table control on managed peatland greenhouse gas emissions. Nature 593, 548–552 (2021).

14. Hambley, G. et al. Net ecosystem exchange from two formerly afforested peatlands undergoing restoration in the Flow Country of northern Scotland. Mires Peat 1–14 (2019) doi:10.19189/MaP.2018.DW.346.
15. Poyda, A., Reinsch, T., Kluß, C., Loges, R. & Taube, F. Greenhouse gas emissions from fen soils used for forage production innorthern Germany. Biogeosciences 13, 5221–5244 (2016).
16. Beyer, C., Liebersbach, H. & Höper, H. Multiyear greenhouse gas flux measurements on a temperate fen soil used for cropland or grassland. J. Plant Nutr. Soil Sci. 178, 99–111 (2015).
17. Elsgaard, L. et al. Net ecosystem exchange of CO2 and carbon balance for eight temperate organic soils under agricultural management. Agric. Ecosyst. Environ. 162, 52–67 (2012).
18. Maljanen, M., Komulainen, V.-M., Hytönen, J., Martikainen, P. J. & Laine, J. Carbon dioxide, nitrous oxide and methane dynamics in boreal organic agricultural soils with different soil characteristics. Soil Biol. Biochem. 36, 1801–1808 (2004).
19. Fleischer, E., Khashimov, I., Hölzel, N. & Klemm, O. Carbon exchange fluxes over peatlands in Western Siberia: Possible feedback between land-use change and climate change. Sci. Total Environ. 545–546, 424–433 (2016).
20. Keck, H. et al. Setting-aside cropland did not reduce greenhouse gas emissions from a drained peat soil in Sweden. Front. Environ. Sci. 12, (2024).
21. Kandel, T. P., Elsgaard, L. & Lærke, P. E. Measurement and modelling of CO2 flux from a drained fen peatland cultivated with reed canary grass and spring barley. GCB Bioenergy 5, 548–561 (2013).
22. Evans, C. et al. Final report on project SP1210: Lowland peatland systems in England and Wales – evaluating greenhouse gas fluxes and carbon balances. (2016).
23. Strack, M. & Zuback, Y. C. A. Annual carbon balance of a peatland 10 yr following restoration. Biogeosciences 10, 2885–2896 (2013).
24. Beyer, J. C. Greenhouse gas exchange of organic soils in Northwest Germany : effects

of organic soil cultivation, agricultural land use and restoration. Treibhausgas-Austausch organischer Böden in Nordwest-Deutschland : Effekte von Bodenbearbeitung, landwirtschaftlicher Nutzung und Wiedervernässung organischer Böden https://media.suub.uni-bremen.de/handle/elib/712 (2014).

25. Salm, J.-O. et al. Emissions of CO2, CH4 and N2O from undisturbed, drained and mined peatlands in Estonia. Hydrobiologia 692, 41–55 (2012).
26. Mander, Ü. et al. Reed canary grass cultivation mitigates greenhouse gas emissions from abandoned peat extraction areas. GCB Bioenergy 4, 462–474 (2012).
27. Adkinson, A. C., Syed, K. H. & Flanagan, L. B. Contrasting responses of growing season ecosystem CO2 exchange to variation in temperature and water table depth in two peatlands in northern Alberta, Canada. J. Geophys. Res. Biogeosciences 116, (2011).
28. Heiskanen, L. et al. Carbon dioxide and methane exchange of a patterned subarctic fen during two contrasting growing seasons. Biogeosciences 18, 873–896 (2021).
29. Aurela, M. et al. Carbon dioxide exchange on a northern boreal fen. Boreal Environ. Res. 14, 699–710 (2009).
30. Pullens, J. W. M., Sottocornola, M., Kiely, G., Toscano, P. & Gianelle, D. Carbon fluxes of an alpine peatland in Northern Italy. Agric. For. Meteorol. 220, 69–82 (2016).
31. Tong, C. H. M. et al. The Carbon Balance of a Rewetted Minerogenic Peatland Does Not Immediately Resemble That of Natural Mires in Boreal Sweden. Glob. Change Biol. 31, e70169 (2025).
32. Peacock, M. et al. The full carbon balance of a rewetted cropland fen and a conservation-managed fen. Agric. Ecosyst. Environ. 269, 1–12 (2019).
33. Olson, D. M., Griffis, T. J., Noormets, A., Kolka, R. & Chen, J. Interannual, seasonal, and retrospective analysis of the methane and carbon dioxide budgets of a temperate peatland. J. Geophys. Res. Biogeosciences 118, 226–238 (2013).
34. Offermanns, L. et al. High greenhouse gas emissions after grassland renewal on bog peat soil. Agric. For. Meteorol. 331, 109309 (2023).
35. Shurpali, N. et al. GHG balance, its seasonality and response to soil type and

management of northern agricultural grasslands: Eddy-covariance flux measurements from three adjacent fields in Finland. Agric. Ecosyst. Environ. 393, 109841 (2025).

36. Schrier-Uijl, A. P. et al. Agricultural peatlands: towards a greenhouse gas sink – a synthesis of a Dutch landscape study. Biogeosciences 11, 4559–4576 (2014).
37. Buzacott, A. J. V. et al. A Bayesian inference approach to determine experimental Typha latifolia paludiculture greenhouse gas exchange measured with eddy covariance. Agric. For. Meteorol. 356, 110179 (2024).
38. Aben, R. C. H. et al. CO2 emissions of drained coastal peatlands in the Netherlands and potential emission reduction by water infiltration systems. Biogeosciences 21, 4099–4118 (2024).
39. Paul, S., Ammann, C., Alewell, C. & Leifeld, J. Carbon budget response of an agriculturally used fen to different soil moisture conditions. Agric. For. Meteorol. 300, 108319 (2021).
40. Aslan-Sungur, G., Lee, X., Evrendilek, F. & Karakaya, N. Large interannual variability in net ecosystem carbon dioxide exchange of a disturbed temperate peatland. Sci. Total Environ. 554–555, 192–202 (2016).
41. Byun, E. et al. Temperature, moisture and freeze–thaw controls on CO2 production in soil incubations from northern peatlands. Sci. Rep. 11, 23219 (2021).
42. D′Acunha, B., Morillas, L., Black, T. A., Christen, A. & Johnson, M. S. Net Ecosystem Carbon Balance of a Peat Bog Undergoing Restoration: Integrating CO2 and CH4 Fluxes From Eddy Covariance and Aquatic Evasion With DOC Drainage Fluxes. J. Geophys. Res. Biogeosciences 124, 884–901 (2019).
43. Nyberg, M. et al. Impacts of Active Versus Passive Re-Wetting on the Carbon Balance of a Previously Drained Bog. J. Geophys. Res. Biogeosciences 127, e2022JG006881 (2022).
44. Satriawan, T. W. et al. Interannual variability of carbon dioxide (CO2) and methane (CH4) fluxes in a rewetted temperate bog. Agric. For. Meteorol. 342, 109696 (2023).
45. Lee, S.-C. et al. Annual greenhouse gas budget for a bog ecosystem undergoing

restoration by rewetting. Biogeosciences 14, 2799–2814 (2017).

46. Lafleur, P. M., Roulet, N. T., Bubier, J. L., Frolking, S. & Moore, T. R. Interannual variability in the peatland-atmosphere carbon dioxide exchange at an ombrotrophic bog. Glob. Biogeochem. Cycles 17, (2003).

47. McVeigh, P., Sottocornola, M., Foley, N., Leahy, P. & Kiely, G. Meteorological and functional response partitioning to explain interannual variability of CO2 exchange at an Irish Atlantic blanket bog. Agric. For. Meteorol. 194, 8–19 (2014).

48. Behrens, N., Knorr, K.-H., Rückriem, C. & Gharun, M. The timing of warming matters as much as its intensity for the annual carbon balance of a degraded raised bog. Biogeosciences 23, 5071–5094 (2026).

49. Hurkuck, M., Brümmer, C. & Kutsch, W. L. Near-neutral carbon dioxide balance at a seminatural, temperate bog ecosystem. J. Geophys. Res. Biogeosciences 121, 370–384 (2016).

50. Schaller, C., Hofer, B. & Klemm, O. Greenhouse Gas Exchange of a NW German Peatland, 18 Years After Rewetting. J. Geophys. Res. Biogeosciences 127, (2022).

51. Alekseychik, P. et al. Carbon balance of a Finnish bog: temporal variability and limiting factors based on 6 years of eddy-covariance data. Biogeosciences 18, 4681–4704 (2021).

52. Lhosmot, A. et al. Biotic and Abiotic Control Over Diurnal CH4 Fluxes in a Temperate Transitional Poor Fen Ecosystem. Ecosystems 26, 951–968 (2023).

53. Bertrand, G. et al. Statistical hydrology for evaluating peatland water table sensitivity to simple environmental variables and climate changes application to the mid-latitude/altitude Frasne peatland (Jura Mountains, France). Sci. Total Environ. 754, 141931 (2021).

54. Li, Q., Gogo, S., Leroy, F., Guimbaud, C. & Laggoun-Défarge, F. Response of Peatland CO2 and CH4 Fluxes to Experimental Warming and the Carbon Balance. Front. Earth Sci. 9, (2021).

55. Hendriks, D. M. D., van Huissteden, J. & Dolman, A. J. The full greenhouse gas balance

of an abandoned peat meadow. (2007).

56. Nilsson, M. et al. Contemporary carbon accumulation in a boreal oligotrophic minerogenic mire – a significant sink after accounting for all C-fluxes. Glob. Change Biol. 14, 2317–2332 (2008).

57. Helfter, C. et al. Drivers of long-term variability in CO2 net ecosystem exchange in a temperate peatland. Biogeosciences 12, 1799–1811 (2015).

58. Ratcliffe, J. et al. Contemporary carbon fluxes do not reflect the long-term carbon balance for an Atlantic blanket bog. The Holocene 28, (2017).
