# Supplementary material for: Drivers of northern peatland CO2 fluxes revisited: interacting water level-temperature dependency
Source: Nat Commun. 2026 Sep 4;17:9504. doi: 10.1038/s41467-026-77456-6 (PMC13545207; doi:10.1038/s41467-026-77456-6)
Supplement: Supplementary file 2 [file 41467_2026_77456_MOESM2_ESM.pdf]

Description of Additional Supplementary Information

Table 1: Peatland sites used for the explainable machine learning analysis of annual CO2 fluxes
