# Supplementary material for: Drivers of northern peatland CO2 fluxes revisited: interacting water level-temperature dependency
Source: Nat Commun. 2026 Sep 4;17:9504. doi: 10.1038/s41467-026-77456-6 (PMC13545207; doi:10.1038/s41467-026-77456-6)
Supplement: Supplementary file 3 [file 41467_2026_77456_MOESM4_ESM.pdf]

nature portfolio

Peer Review File

Drivers of northern peatland CO2 fluxes revisited: interacting water level-temperature dependency

Corresponding Author: Mr Nicolas Behrens

This manuscript has been previously reviewed at another journal. This document only contains information relating to versions considered at Nature Communications.

This file contains all reviewer reports in order by version, followed by all author rebuttals in order by version.

Version 0:

Reviewer comments:

Reviewer #1

(Remarks to the Author)
I have read the re-revised manuscript transferred to Nature Communications. The authors have taken the additional comments into consideration, and the revision is further improved. I do not have any further comments.

(Remarks on code availability)

Reviewer #2

(Remarks to the Author)
I appreciate the time and effort that the authors have put into their revisions. They have addressed my original comments and concerns, and have clarified the text and their findings accordingly. I do not have further comments.

(Remarks on code availability)

Reviewer #3

(Remarks to the Author)
The study analyzes a compilation of annual and daily CO_2 exchange rates in temperate and boreal regions and zooms in to study how temperature, water table and their interaction regulate the flux. The result that water table dependence is nonlinear and that the combined effect of temperature and water table differs between cold and warm sites is a valuable and timely contribution in the field. The authors have done an excellent job in clarifying many concerns raised by the previous reviewers, particularly in making the dataset more balanced across different water table depths and land cover types in the machine learning analysis. They have also been careful with their interpretations and conclusions that now are mostly in line with the strengths and limitations of the data. I have a few suggestions to further improve the manuscript, which are listed below.

I think the authors should provide a short justification for excluding forestry drained peatlands from their dataset. Forestry is mentioned in the introduction as one of the key land use types in peatlands and there is plenty of both EC and chamber data from such sites, exactly in the water level range of 60-100 cm the authors now write has a shortage of data in the boreal region.

In the analysis of daily data it was found that site identity was the most important predictor, followed by SWIN and LAI. I wonder if the authors have tried to include land cover type as a predictor here like they did with the annual model. Land cover type may not be as strong predictor as site, but would contain much more information for future studies, namely about the differences between site types (in this subset of data mostly fens and bogs). One could even examine the interactions of site type with other predictors and find out if the responses to other variables differ between site types.

Line-by-line comments:

L75-76 Vegetation activity means leaf area here, suggest writing that instead.

L77-79 Write already here (or the following paragraph) that you only included treeless sites.

L98 I suggest reconsidering the terminological choice to call the key response variable as net biome exchange. I find it misleading as biome has another meaning, and it doesn't really help to understand that this is NECB excluding DOC and methane.

Fig. I believe this is the first time the abbreviation SHAP is mentioned, please explain it. Perhaps also very shortly how it is interpreted.

L186-187 In fact all other predictors have rather similar importance compared to WTD.

L200-205 Do you have any interpretation for this response shape and threshold? Perhaps temperature enhances plant productivity at low temperatures, but respiration becomes more important at higher temperatures as it has an exponential response to temperature? I know the data cannot answer this directly, but perhaps some interpretation would be possible.

Extended Data Table 2: Please indicate in the caption that this was the data for daily analysis.

Extended Data Fig. 1: Would it be possible to have also a boxplot with the emissions of the colder and warmer subsets (maybe by land cover type without the method separation)? There could also be a mean across the entire dataset marked to facilitate the interpretation of the SHAP values. Currently the colder and warmer subset are only presented through the SHAP values.

Extended Data Fig. 6 and L221-244: In this graph the individual datapoints are a result of resampling 20 times. Displaying all these resampled points may obscure the fact that in the colder subset at WT greater than about 50-60 cm there are only very few sites, and they are all managed. Most of these sites display SHAP-TA interaction close to zero (or even positive). Then there are two datapoints (both from Tyumen) that have a highly negative interaction and very low WT. I think this one year from Tyumen is the only reason the authors see a negative interaction at colder sites, which would otherwise be close to zero. The authors are correct in mainly interpreting the positive interaction at the warmer sites, which is backed up by a larger quantity of data. However, I would be even more careful than currently in interpreting the negative interaction at the colder sites. Firstly, I suggest the authors present in this graph the original datapoints, and the points from 20 resampling could be plotted with faded color in the background. Secondly, I suggest modifying the text on L221-244 so that the very high uncertainty related to the negative interaction at colder sites becomes directly evident.

L264-266 Is this not just a complex way to say that temperature increases emissions? Perhaps it would be better to interpret the magnitude of SHAP values in different seasons. Furthermore, the timing of winter, spring and autumn are very different in the included sites. I suggest using months instead. For example, positive SHAP values extend until November, when there is already snow cover in many sites.

(Remarks on code availability)

Version 1:

Reviewer comments:

Reviewer #3

(Remarks to the Author)
I have read the re-revised version of the manuscript. I appreciate the effort the authors have made to address the comments. I think in the present state the study is very well balanced in interpretations. Thank you also for displaying the suggested analysis in the response letter even though they did not provide material for the manuscript itself. I do not have any further comments.

(Remarks on code availability)

Open Access This Peer Review File is licensed under a Creative Commons Attribution 4.0 International License, which permits use, sharing, adaptation, distribution and reproduction in any medium or format, as long as you give appropriate credit to the original author(s) and the source, provide a link to the Creative Commons license, and indicate if changes were made.
In cases where reviewers are anonymous, credit should be given to 'Anonymous Referee' and the source.
The images or other third party material in this Peer Review File are included in the article's Creative Commons license, unless indicated otherwise in a credit line to the material. If material is not included in the article's Creative Commons license and your intended use is not permitted by statutory regulation or exceeds the permitted use, you will need to obtain permission directly from the copyright holder.
To view a copy of this license, visit https://creativecommons.org/licenses/by/4.0/

Title: "Drivers of northern peatland CO_2 fluxes revisited: interacting water level-temperature dependency"

Behrens et al.

21 July 2026

We thank the handling editor for the opportunity to submit a revised version of our manuscript "Drivers of northern peatland CO_2 fluxes revisited: interacting water level-temperature dependency".

In the following is our point-by-point response to the comments of 3 reviewers. Reviewer #1 and #2 have praised our study and requested no changes. Reviewer #3 raised points that helped to improve the strength of our discussion and to increase the clarity of the text. We have addressed every comment thoroughly.

In summary:

- we added additional justifications proposed by Reviewer #3 to exclude forest sites and provided further references as to why we chose NBE as the primary response variable name
- we added a brief discussion of whether our data supports a distinction of emission factors (or NBE budgets) between climate regions. We adjusted our interpretation of the ML derived effect of TA to a partial effect of this variable while accounting for other drivers, rather than a clear threshold for different average emissions.
- we adjusted the text regarding the interactive TA-WTDe effect on annual NBE to more clearly convey the uncertainty related to data scarcity in boreal regions.
- we adjusted the discussion of the seasonality of the TA effect on daily CO_2 fluxes to be more nuanced, which now describes the dynamics more precisely and is well in line with previous research.
- we implemented an improved validation procedure for the daily scale analysis, testing the model on fully left out years, preventing any data leakage between train and test data. While not changing the driver dynamics, this approach provides a more solid metric of the model performance.

We invite you to consult our detailed, point-by-point response to the reviewers' comments in the following and thank you for your time.

[The Authors´ responses are marked in blue. Line numbers correspond to the version with tracked changes.]

Reviewer #1:

I have read the re-revised manuscript transferred to Nature Communications. The authors have taken the additional comments into consideration, and the revision is further improved. I do not have any further comments.

Reviewer #2:

I appreciate the time and effort that the authors have put into their revisions. They have addressed my original comments and concerns, and have clarified the text and their findings accordingly. I do not have further comments.

Reviewer #3:
The study analyzes a compilation of annual and daily CO2 exchange rates in temperate and boreal regions and zooms in to study how temperature, water table and their interaction regulate the flux. The result that water table dependence is nonlinear and that the combined effect of temperature and water table differs between cold and warm sites is a valuable and timely contribution in the field. The authors have done an excellent job in clarifying many concerns raised by the previous reviewers, particularly in making the dataset more balanced across different water table depths and land cover types in the machine learning analysis. They have also been careful with their interpretations and conclusions that now are mostly in line with the strengths and limitations of the data. I have a few suggestions to further improve the manuscript, which are listed below.

Response: We thank the reviewer for recognizing and acknowledging our work

I think the authors should provide a short justification for excluding forestry drained peatlands from their dataset. Forestry is mentioned in the introduction as one of the key land use types in peatlands and there is plenty of both EC and chamber data from such sites, exactly in the water level range of 60-100 cm the authors now write has a shortage of data in the boreal region

Response: We agree with the reviewer and have provided a justification and additional text in lines 81-83. The text now reads:
"Peatlands drained for forestry were excluded as plantation cycles span over several decades and carbon cycles are not considered to be in a steady state (Evans et al. 2021)."

In the analysis of daily data it was found that site identity was the most important predictor, followed by SWIN and LAI. I wonder if the authors have tried to include land cover type as a predictor here like they did with the annual model. Land cover type may not be as strong predictor as site, but would contain much more information for future studies, namely about the differences between site types (in this subset of data mostly fens and bogs). One could even examine the interactions of site type with other predictors and find out if the responses to other variables differ between site types.

Response: We thank the reviewer for this suggestion. We tested how the replacement of the site ID with land-cover type as categorical variable affects the model and whether the responses to other drivers vary across land-cover types. The results however didn't justify expanding the analysis in this direction.
After replacing site-ID with land-cover type in the analysis of daily data, the validation R^2 dropped to 0.53, and RMSE increased by 37% from 2.4 to 3.3. Both is expected as the model becomes much less flexible. Notably the driver dynamics based on SHAP values did not change between the site types (bog, fen, extraction). Only the extraction site fluxes reacted weaker to increasing SWIN and LAI, which is well expected due to the sparse vegetation cover. Given that this test did not reveal new patterns, we have kept the analysis as before. Please however, see the results of this test below. Note that with the newly implemented cross-validation (now testing the model on fully left out site-years) the site ID

became the second most important predictor, followed by radiation. However, these two are only a few percent apart, both with the former and the new model.

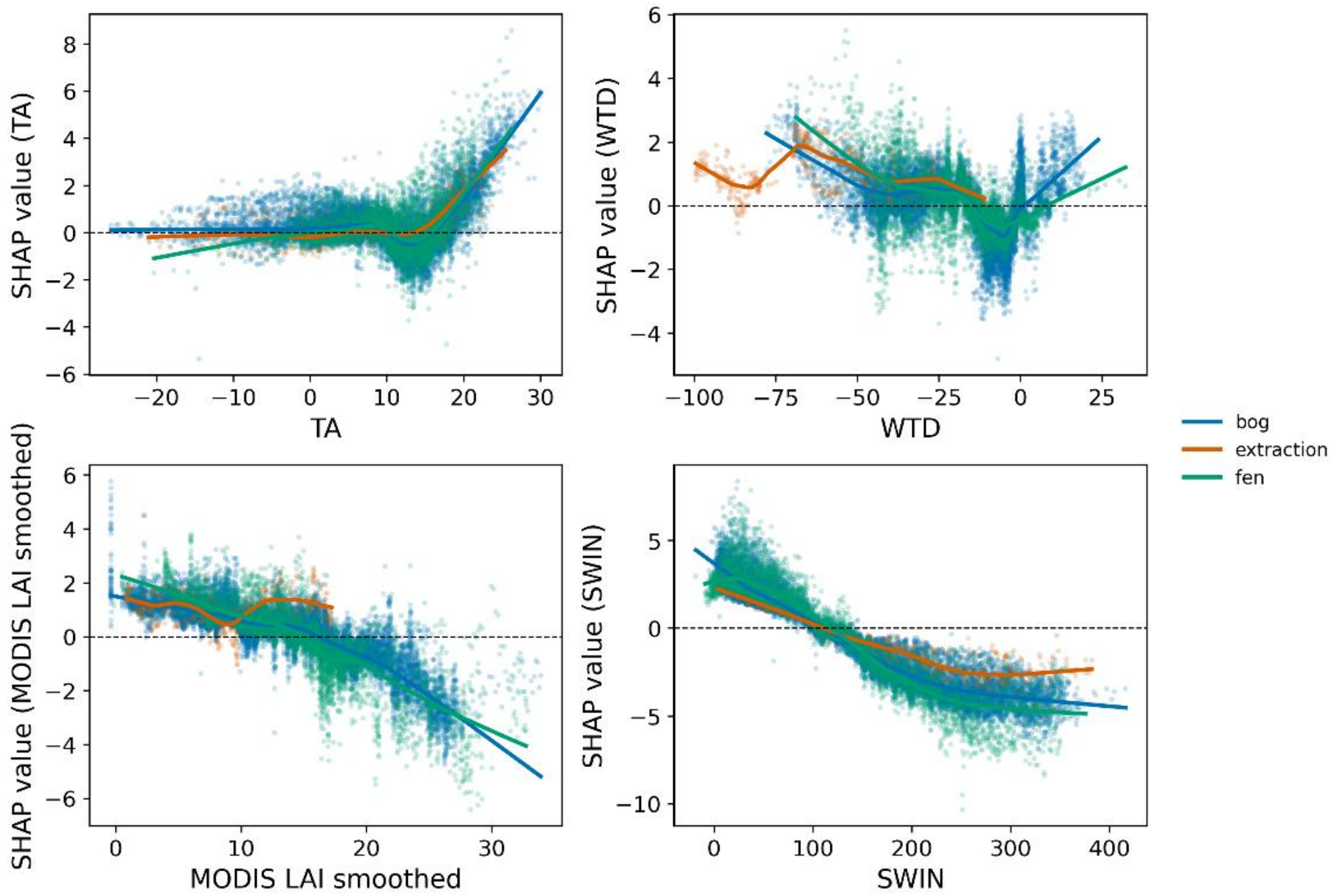

Line-by-line comments:

L75-76 Vegetation activity means leaf area here, suggest writing that instead.

Response: We agree and incorporated this change (lines 76-77).

L77-79 Write already here (or the following paragraph) that you only included treeless sites.

Response: We agree and incorporated this change (lines 81-83).

L98 I suggest reconsidering the terminological choice to call the key response variable as net biome exchange. I find it misleading as biome has another meaning, and it doesn't really help to understand that this is NECB excluding DOC and methane.

Response: We have followed the definition of the "net biome exchange" (or its inverse "net biome production") by Schulze et al. (2000) and Kirschbaum et al. (2001), as it incorporates the included components of GPP, respiration fluxes and harvest losses. NECB describes the carbon balance including DOC and CH_4 in contrast to e.g., NEP or NEE, which is why we refrain from using this term.
We added two references in line 102 in the explanation of NBE to support our choice of terminology.

Fig. 3 I believe this is the first time the abbreviation SHAP is mentioned, please explain it. Perhaps also very shortly how it is interpreted.

Response: While we already had included an explanation of how to interpret SHAP values in the caption of figure 3, we added to it for additional clarification as follows:

"Figure 3. **Effects of WTDe and TA on peatland NBE derived through explainable machine learning.** Effects of WTDe (**a**) and TA (**b**) on annual NBE budgets assessed with SHAP values. SHAP values are a measure of driver strengths in machine learning models. Positive/negative SHAP values indicate an increasing/decreasing effect of the driver on NBE, while values near zero indicate a weak effect. The red dashed lines are LOESS functions, representing a smoothed response curve."

186-187 In fact all other predictors have rather similar importance compared to WTD.

Response: That is correct. We have made this point explicit now by reformulating lines 196-200 as:
"WTDe was the main predictor with 43% ± 2% of the feature importance, followed by TA (17% ± 1.7%) SWIN (15% ± 1.4%), LAI (13% ± 1.2%) and the site ID (13% ± 1.4%) with similar contributions (Extended Data Fig. 4 a and b)."

L200-205 Do you have any interpretation for this response shape and threshold? Perhaps temperature enhances plant productivity at low temperatures, but respiration becomes more important at higher temperatures as it has an exponential response to temperature? I know the data cannot answer this directly, but perhaps some interpretation would be possible.

Response: We thank the reviewer for this suggestion. We have added an interpretation of the response shape in lines 236-243, highlighting that the machine learning approach cannot directly provide mechanistic insights:

"Because the machine-learning analysis identifies statistical relationships rather than causal mechanisms, and because the data represents annual-scale fluxes, the processes underlying the transition in TA effects cannot be directly resolved. However, a possible explanation for the shift is that warmer conditions enhance temperature-sensitive respiration more strongly than carbon uptake processes, especially under dry conditions[43,44]. As ca. 50% of the sites have a mean annual WTDe of more than 30 cm this may explain that a higher mean annual temperature predominantly led to a greater contribution of respiration-driven carbon losses at warmer sites."

Extended Data Table 2: Please indicate in the caption that this was the data for daily analysis.

Response: The requested change was made (lines 1368-1369).

Extended Data Fig. 1: Would it be possible to have also a boxplot with the emissions of the colder and warmer subsets (maybe by land cover type without the method separation)? There could also be a mean across the entire dataset marked to facilitate the interpretation of the SHAP values. Currently the colder and warmer subset are only presented through the SHAP values.

Response: We checked the distribution of mean annual CO_2 budgets in the warmer and colder sites and found no significant difference within the land-cover types between the TA regimes. We included this limitation in the discussion of the detected TA effect. We now strictly interpret the TA response as a nonlinear contribution of TA to CO_2 fluxes combined

with the other drivers instead of a fixed threshold separating groups of budgets (or emission factors). We phrased it as follows in lines 216-221:

"We found a non-linear transition of the TA effect between sites with temperatures in the range of ca. 4 and 8 °C and those with higher temperatures. At the colder sites, the effect of TA on modeled NBE was small or negative. At temperatures above 8 °C the effect shifted towards a scattered but predominantly positive, NBE increasing effect, shown by largely positive SHAP values of TA."

Given that the differences were non-significant (*p* > 0.05) we did not add an additional boxplot, however the boxplot is shown below this response. We have included this discussion point now in the text, in lines: 225-232:

"Based on this finding we tested whether NBE within the landcover types differed between the colder and warmer temperature regimes, as suggested e.g. in the IPCC Tier 1 emission factors for boreal and temperate natural peatlands[42]. The differences were non-significant for all land-cover types. The transition in SHAP-based TA effects thus rather reflects a non-linear dependency of NBE on temperature specifically after accounting for SWIN, LAI and WTDe as well as site-specific differences rather than a distinction of NBE budgets based on TA thresholds alone."

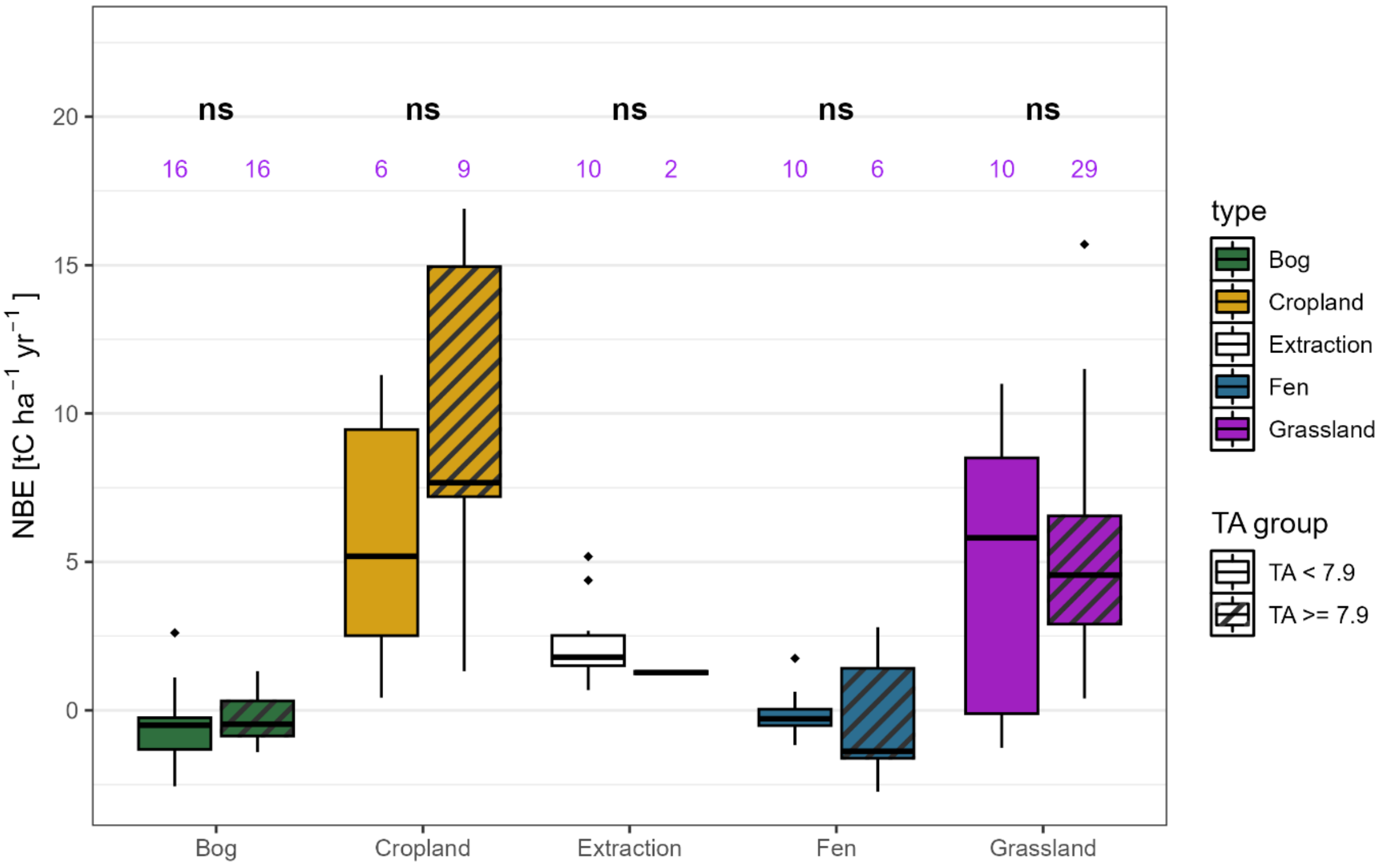

Extended Data Fig. 6 and L221-244: In this graph the individual datapoints are a result of resampling 20 times. Displaying all these resampled points may obscure the fact that in the colder subset at WT greater than about 50-60 cm there are only very few sites, and they are all managed. Most of these sites display SHAP-TA interaction close to zero (or even positive). Then there are two datapoints (both from Tyumen) that have a highly negative interaction and very low WT. I think this one year from Tyumen is the only reason the authors see a negative interaction at colder sites, which would otherwise be close to zero. The authors are correct in mainly interpreting the positive interaction at the warmer sites, which is

backed up by a larger quantity of data. However, I would be even more careful than currently in interpreting the negative interaction at the colder sites. Firstly, I suggest the authors present in this graph the original datapoints, and the points from 20 resampling could be plotted with faded color in the background. Secondly, I suggest modifying the text on L221-244 so that the very high uncertainty related to the negative interaction at colder sites becomes directly evident.

Response: We thank the reviewer for this suggestion. Following the suggestion of the reviewer we have updated Extended Data Figure 6 and improved the Discussion (in lines 253-284) and the Conclusion (in lines 375-378).

We agree that there is substantial uncertainty in the interaction effects, both in the positive and negative range. The interaction effect at several site-years of the TA range >7.9 °C is negative (at ca. 50 cm WTDe) while one grassland (Lavassaare Fen Meadow) of the lower TA range exhibits a positive interaction effect. Notably, the divergent effect is already emerging before, in the range of 20 to 50 cm WTDe. We agree that the real distribution of datapoints should be made more visible. While there is no "true" datapoint, as the points are all results of model outputs based on resampled datasets and thus equally "true", we have added the average of all resamples as a solid datapoint while including all resampled datapoints as faint markers. Further, we fit one LOESS line on each resampling and show the average of all LOESS fits in thick, with the individual lines shown faded.

Further, we moved the mention of the low density of datapoints in the cold temperature regime right behind the discussion of the interaction effect. We rephrased the paragraph to put less emphasis on a clear separation into two regimes but rather focus on an increasing interaction effect with lower WTDe. While we retain the mention of the apparent distinction by temperature, we now specifically point to the gap in data coverage. The rephrased paragraph in lines 253-284 now reads as follows:

"Finally, we looked more closely into the interaction effect of TA and WTDe on NBE.We determined the TA-WTDe interaction effect on a dataset with balanced land-cover types, equally representing site types both in the two TA regimes (above and below 7.9 °C) and along the range of WTDe (see Methods). We found an emergent interaction effect that became stronger with increasing WTDe both in the lower and the higher TA class. At sites with warmer mean annual TA (> 7.9 °C) the interaction term increased from near zero to a predominantly positive effect at WTDe deeper than 30 cm (Extended Data Fig. 6). While there is substantial scatter in the interaction effect in the range of WTDe between 30 and 60 cm within the warmer TA class, the smoothed interaction and its confidence interval remained almost exclusively positive at WTDe of more than ca. 45 cm. This indicates that the combined effect of deeper WTDe and warm site conditions amplifies emissions beyond each individual effect. While to our knowledge this pattern has not been reported on the annual and cross-site scale, it is consistent with evidence of an increased response of respiration to water table depth with higher temperatures [41]. Conversely, at cold sites (TA ≤ 7.9 °C) the smoothed interaction effect was predominantly negative at WTDe deeper than 30 cm, suggesting that the WTDe-driven emission response may be attenuated or partially suppressed at sites with colder temperatures (Extended Data Fig. 6). However, it should be noted that the sample size of deeply drained grassland and cropland sites in the boreal region is very limited with only seven site-years of TA < 7.9°C and WTDe > 50 cm, resulting in a high uncertainty of the interaction effect at deep WTDe and low TA. (Extended Data Fig.

6).
The patterns emerging both from the TA effect alone as well as the TA-WTDe interaction effect point towards a control of the mean annual temperature on CO_2 fluxes. However, to consolidate such general patterns and use them for temporal and spatial projections prevailing gaps in the data need to be closed. Measurement networks such as the Dutch NOBV (Nationaal Onderzoeksprogramma Broeikasgassen Veenweiden) or the UK GHG Flux Network should be established in regions and land-cover types that are currently underrepresented, such as boreal grass- and croplands."

We further rephrased the conclusion in lines 375-378 as:

"At colder sites the effect appeared reversed but the uncertainty remained high due to data scarcity. This indicates that responses of annual peatland CO_2 emissions to hydrology and climate are interconnected and potentially not uniform across climate regions, with implications for how emissions are extrapolated across peatlands differing in mean annual temperature."

Finally we incorporated the more careful phrasing in mention of the TA effect in the abstract to describe both the direct TA effect as well as the interaction effect in lines 30-36 as:

"Across peatlands spanning diverse land-cover—including natural fens and bogs, croplands, grasslands, and extraction sites—CO_2 emissions exhibit a non-linear response to water table depth. Emissions decline when water tables are raised above 60-75 cm depth. Optimal mitigation requires water tables of 20 cm or higher. We find that deep water tables interact with temperatures to increase emissions at temperate sites."

L264-266 Is this not just a complex way to say that temperature increases emissions? Perhaps it would be better to interpret the magnitude of SHAP values in different seasons. Furthermore, the timing of winter, spring and autumn are very different in the included sites. I suggest using months instead. For example, positive SHAP values extend until November, when there is already snow cover in many sites.

Response: We thank the reviewer for this suggestion. We have rephrased our text (in lines 323-334) to provide a more nuanced presentation of the magnitude of SHAP values in different seasons and reflect the suggestion of the reviewer. The discussion now reads:

"The effect of TA changes over the course of the year. Between mid-November and mid-February low temperatures have little impact on CO_2 fluxes. Between mid-February and the beginning of April the effect of TA rises to an emission-increasing effect, likely depicting the impact of increasing temperatures on respiration before the onset of vegetation activity. Notably, between the start of April and the middle of June this TA effect continuously reduces until it is nearly zero, around the same time the effect of LAI becomes most negative (thus uptake enhancing). Afterwards, in mid- to late summer, the model attributed an emission-enhancing effect to higher temperatures (Fig. 4). This pattern aligns with a previous study which found that warming induced increasing CO_2 emissions in late summer and autumn and increased uptake in spring and early summer[46]."

References:

Evans, C. D. et al. Overriding water table control on managed peatland greenhouse gas emissions. Nature 593, 548–552 (2021).

Schulze, E.-D., Wirth, C. & Heimann, M. Managing Forests After Kyoto. Science 289, 2058–2059 (2000).

Kirschbaum, M., Eamus, D., Gifford, R., Roxburgh, S. & Sands, P. Definitions of some ecological terms commonly used in carbon accounting. Cooperative Research Centre for Carbon Accounting, Canberra 2–5 (2001).
